# Constructing care cascades for active tuberculosis: A strategy for program monitoring and identifying gaps in quality of care

**DOI:** 10.1371/journal.pmed.1002754

**Published:** 2019-02-27

**Authors:** Ramnath Subbaraman, Ruvandhi R. Nathavitharana, Kenneth H. Mayer, Srinath Satyanarayana, Vineet K. Chadha, Nimalan Arinaminpathy, Madhukar Pai

**Affiliations:** 1 Department of Public Health and Community Medicine and Center for Global Public Health, Tufts University School of Medicine, Boston, Massachusetts, United States of America; 2 Division of Geographic Medicine and Infectious Diseases, Tufts Medical Center, Boston, Massachusetts, United States of America; 3 Division of Infectious Diseases, Beth Israel Deaconess Medical Center and Harvard Medical School, Boston, Massachusetts, United States of America; 4 The Fenway Institute, Boston, Massachusetts, United States of America; 5 Centre for Operational Research, International Union Against Tuberculosis and Lung Disease, Paris, France; 6 Central Leprosy Teaching and Research Institute, Chengalpattu, India; 7 MRC Centre for Global Infectious Disease Analysis, School of Public Health, Imperial College London, London, United Kingdom; 8 Department of Epidemiology, Biostatistics and Occupational Health and McGill International TB Centre, McGill University, Montreal, Canada

## Abstract

The cascade of care is a model for evaluating patient retention across sequential stages of care required to achieve a successful treatment outcome. This approach was first used to evaluate HIV care and has since been applied to other diseases. The tuberculosis (TB) community has only recently started using care cascade analyses to quantify gaps in quality of care. In this article, we describe methods for estimating gaps (patient losses) and steps (patients retained) in the care cascade for active TB disease. We highlight approaches for overcoming challenges in constructing the TB care cascade, which include difficulties in estimating the population-level burden of disease and the diagnostic gap due to the limited sensitivity of TB diagnostic tests. We also describe potential uses of this model for evaluating the impact of interventions to improve case finding, diagnosis, linkage to care, retention in care, and post-treatment monitoring of TB patients.

## Abstract 2

In a Guidelines and Guidance article, Ramnath Subbaraman and colleagues advise on using care cascade analyses to assess programs for active tuberculosis treatment.

## Introduction

Tuberculosis (TB) is the leading infectious cause of death globally [1]. The World Health Organization (WHO) has highlighted “patient-centered care for all people with TB” as a central pillar of its post-2015 End TB strategy [2]. The cascade of care (also called the continuum of care) is a useful model for evaluating patient retention across sequential stages of care required to achieve a successful outcome. The cascade helps to quantify gaps in care delivery, pointing to areas in which quality of care could be improved. Over the last decade, the HIV community has pioneered use of the cascade to evaluate care delivery in diverse populations [3–5]. This model has subsequently been applied to other diseases [6,7]. The care cascade is instrumental in tracking progress in the Joint United Nations Programme on HIV/AIDS (UNAIDS) 90-90-90 global strategy for HIV [8,9].

Care cascades have only recently been used to evaluate TB care [10,11], although TB programs have a tradition of conducting cohort analyses and, more recently, of using patient pathways analyses to understand dropouts in care [12]. In addition, WHO has outlined an onion model in which patient losses across different steps in care are visualized as a series of concentric circles [13], and this conceptual model informs our approach to the care cascade.

The United Nations Secretary General’s Special Envoy on TB has called for more widespread use of care cascade analyses to help achieve the End TB strategy [14]. In addition, National Strategic Plans for India and South Africa refer to closing gaps in the care cascade as a key component of their TB elimination strategies [15,16]. We discuss approaches for estimating care cascade stages for individuals with active TB, describe uses of this model for targeting interventions to address gaps in care, and suggest areas for future research. We argue that the care cascade has two potential benefits: as an approach for quantifying TB outcomes and as a conceptual framework for examining the quality of health services across various stages of care.

TB has a range of states, ranging from latent infection (in which bacilli lie dormant, controlled by the immune system) to subclinical disease (in which the patient has no symptoms but has microbiological or radiographic evidence of disease) to active disease (in which the patient has symptoms in addition to microbiological or radiographic findings) [17]. The current manuscript describes an approach for estimating the care cascade for active disease. We do not cover treatment of latent infection, which affects around one-quarter of the world’s population [18]. Other articles provide guidance on constructing care cascades for TB subpopulations, including individuals with latent infection [19], children with active disease [20], individuals with HIV/TB coinfection [21], and household contacts of TB patients [22].

### A model for the TB care cascade, with examples from India and South Africa

**Fig 1:**
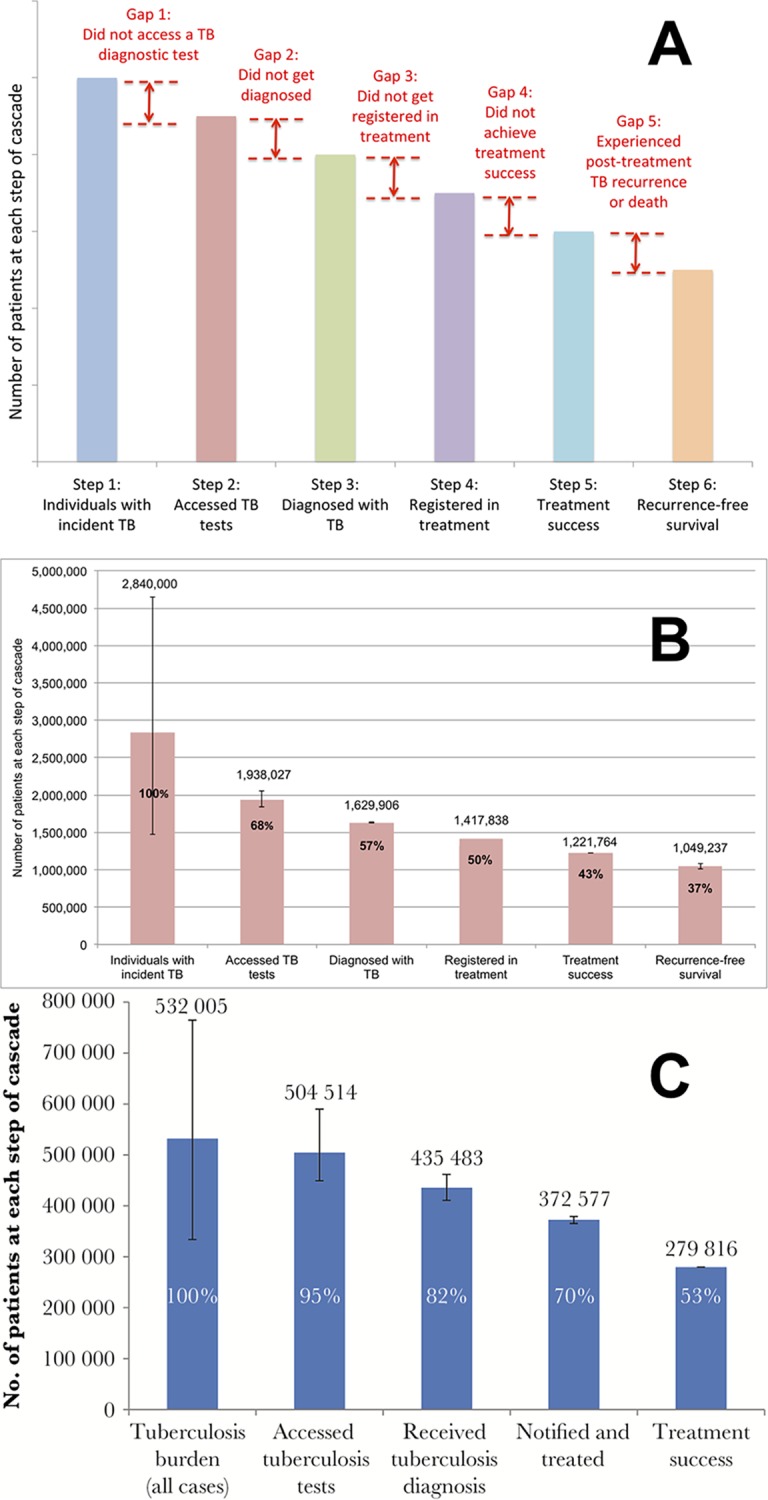
Examples of TB care cascades, including a generic model. (A) A generic model for a care cascade for active TB; (B) the care cascade for individuals with any form of active TB in India in 2013, modified from [10] based on updated WHO TB incidence estimates [23]; and (C) the care cascade for patients with any form of active TB in South Africa in 2013 [11]. The Indian care cascade has 1-year recurrence-free survival as the final step, while the South African care cascade stops at treatment success. Individuals with latent TB are not included in these models. Whiskers represent 95% confidence intervals. TB, tuberculosis; WHO, World Health Organization.

In Fig 1 (panel A), we present a model for the TB care cascade, integrating the WHO onion model with elements of the HIV care cascade [10,13]. Each cascade stage contains a step (i.e., the absolute number of individuals achieving a point in care) and a gap (i.e., the difference between steps, representing individuals with suboptimal outcomes). Recent studies in India and South Africa used this general approach to estimate national-level TB outcomes. These countries differ with regard to HIV prevalence, initial diagnostic tests used, and healthcare landscape (Table 1) [10,11]. The studies presented outcomes for 2013 despite being published in 2016 and 2017, respectively, because multidrug-resistant tuberculosis (MDR TB) outcomes take 3 years to be reported, given the long treatment duration.

**Table 1:** Comparison of the Indian and South African TB care cascades for 2013.

	Indian TB care cascade (modified from [10])^a^	South African TB care cascade (from [11])
**Country context**		
**Epidemiology**	Low HIV prevalence	High HIV prevalence
**Healthcare landscape**	Similar proportions of TB patients are treated in the private and public sector	Public sector treats the vast majority of TB patients
**Most common tests used to diagnose TB**	Sputum microscopy as the most common frontline test	Xpert MTB/RIF and sputum microscopy as the frontline tests
**Methodological approach for constructing the cascade**		
**Data sources**	Number of treated patients from country TB reports; meta-analyses of local studies to estimate key gaps	Number of diagnosed and treated patients from a national electronic TB register; meta-analysis of local studies to estimate PTLFU
**Total number of individuals with TB at the population level**	Estimated number of prevalent TB cases in 2013 (modified Fig 1 uses revised WHO TB incidence estimates for India [23])	Estimated number of incident TB cases in 2013 plus half of the estimated number of patients with undetected TB in 2012
**Choice of end outcome for the cascade**	1-year recurrence-free survival	Treatment success^b^
**Study findings**		
**Care cascade completion rate for all forms of TB**^c^	43%^a^^,^^c^	53%
**Care cascade completion rate for MDR TB**^c^	7%^a^^,^^c^	22%

^a^These estimates are adjusted from the original publication based on revised TB incidence estimates for India in 2015. Overall TB incidence in India was revised substantially upward by WHO, and estimates of MDR TB incidence in India were not available in prior WHO reports.

^b^Treatment success is defined as patients who either achieved cure or treatment completion.

^c^Cascade completion here is defined as the outcome of treatment success, rather than recurrence-free survival to allow comparison between the Indian and South African cascades.

**Abbreviations:** MDR TB, multidrug-resistant TB; PTLFU, pretreatment loss to follow-up; TB, tuberculosis.

**Fig 2:**
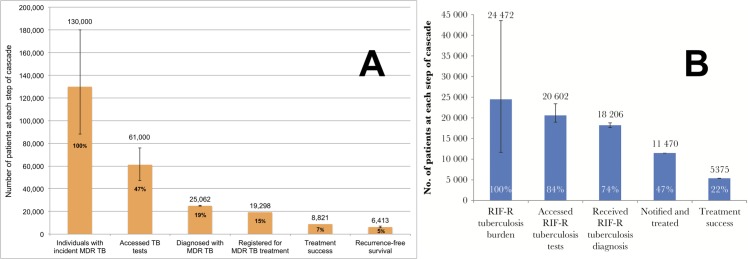
Examples of MDR TB care cascades. (A) The care cascade for individuals with MDR TB in India in 2013, modified from [10] based on updated WHO MDR TB incidence estimates [23], and (B) the care cascade for individuals with rifampin-resistant TB in South Africa in 2013 [11]. Rifampin resistance is considered to be a surrogate marker for multidrug resistance. The Indian care cascade has 1-year recurrence-free survival as the final step, while the care cascade for South Africa stops at treatment success. Whiskers represent 95% confidence intervals. MDR, multidrug-resistant TB; TB, tuberculosis; WHO, World Health Organization.

Outcomes and major gaps in each country cascade vary, highlighting different deficiencies in care (Figs 1 and 2 and Table 1). The South African program performed better in terms of individuals with TB in the population accessing a TB test (Gap 1) but achieved poorer treatment outcomes than India’s public sector. About 37% of all patient losses in the South African cascade consisted of individuals who experienced poor outcomes during therapy (Gap 4). In contrast, India’s TB program did a poorer job of case finding: 50% of all patient losses consisted of individuals with incident TB who did not access a TB test (Gap 1). For both countries, Gap 2 is the second largest contributor to patient losses. MDR TB cascade outcomes in both countries are very poor, with deficiencies at every stage [10,11].

These two studies may provide insights into the situation in other countries with similar epidemiological contexts. In addition to focusing on other high–TB-burden countries, future cascade analyses should address high-risk populations in countries with a lower TB burden (e.g., immigrants in Europe) and countries with high MDR TB rates (e.g., former Soviet Bloc countries) [24,25], which are epidemiological contexts not represented in the current literature.

### Methods for designing this guidance document

Members of our team contributed to the recent Indian care cascade analysis [10]. We studied methods used in the South African cascade for further insights [11]. Our prior research is relevant for estimating different cascade stages, including the number of individuals with TB in the population (NA, VC) [26,27], the diagnostic gap (MP, RN) [28–30], pretreatment loss to follow-up (PTLFU; RS, SS, VC, MP) [31–35], and post-treatment disease recurrence (VC) [36]. Our team also includes an expert in the HIV care cascade (KM) [37–40]. Input was incorporated from members of our team by email and in-person discussions. Limitations of the analytical approach are described in the main manuscript and S1 Appendix.

### General principles for constructing a cascade

The approach for constructing a care cascade depends on the assessment’s primary goal, which may include the following: (1) large-scale evaluations for monitoring patient outcomes in national programs or (2) smaller-scale evaluations for identifying gaps in quality of care at the clinic, city, or district levels. Large evaluations may aim to achieve nationally representative estimates of patient outcomes, while smaller-scale evaluations may additionally collect data on process indicators (indicators of quality of care) to enable intervention development.

Different approaches for estimating a care cascade have varied risks of bias [41]. Recently published TB care cascades used data from different patient cohorts to estimate each stage—what we refer to as a routine data approach (S1 Appendix) [10,11,19]. This approach does not account for the patient population’s changing composition at each stage, introducing biases that may carry forward to subsequent stages [41]. In a cohort-based approach, the same individuals are followed through each cascade stage, minimizing risk of bias and achieving higher internal consistency (S1 Appendix) [41]. This approach allows estimation of the transition time of patients across stages, which has implications for disease transmission [5,42]. We encourage use of cohort-based approaches whenever possible, although this approach is more resource intensive. If representative sampling of health facilities is used, it may be feasible to estimate cascade outcomes with reasonable precision using moderate samples even for large countries such as India or China. For example, the Population-based HIV Impact Assessment Project uses primary data collection with representative sampling to estimate the HIV care cascade in several African countries [43].

Another challenge in estimating a TB care cascade is that common diagnostic tests for active TB have relatively low (e.g., sputum microscopy) or higher but imperfect (e.g., Xpert MTB/RIF) sensitivity [44,45]. Xpert MTB/RIF, a polymerase chain reaction (PCR)-based test, has 85% to 92% sensitivity for diagnosing pulmonary TB, including rifampin resistance, compared to 40% to 60% sensitivity for sputum microscopy [44], but most high-burden countries are still reliant on microscopy for detecting active TB. A considerable proportion of TB patients are diagnosed empirically, especially when sputum microscopy is the only test used. In contrast, HIV tests have very high sensitivity and specificity, allowing for accurate identification of HIV-infected individuals who should be followed through subsequent cascade stages. HIV viral load also provides a reliable biological marker of effective treatment. In contrast, the diverse forms of TB (e.g., pulmonary, extrapulmonary, drug resistant) and potential for disease recurrence pose unique challenges for estimating TB care cascades. We therefore recommend approaches for estimating each stage based on the primary diagnostic test used in a given setting and the specific form of TB.

### Strategies for inclusion of private sector TB patients

A challenge for estimating care cascades in many countries (e.g., India [26,46], Indonesia [47], and Pakistan [48]) is that a large proportion of TB patients are managed in the private sector. Notification rates for these patients are low [26,46,49,50]. They are often treated empirically, without undergoing bacteriological testing [51,52], and the quality of private sector care is poor in standardized patient studies [53].

Given low private sector notification rates, representative sampling of private laboratories with TB testing capabilities could allow cohort-based tracking of patients starting from Step 2 (accessed a TB test). Audits of lab registers would identify bacteriologically diagnosed private sector patients who may not be notified to national programs. From Step 2, approaches for estimating cascade stages would be similar to those for the public sector; however, this approach does not account for private sector patients who are diagnosed empirically, without a TB test. As such, representative sampling of private clinics that deliver a high volume of TB care (e.g., qualified physicians participating in public–private mix projects) may also be necessary in settings with high rates of empirical treatment. Chart audits could identify patients at these clinics who are treated empirically, who could be followed for treatment outcomes and disease recurrence rates.

### Estimating each stage of the TB care cascade

We describe approaches for estimating the TB care cascade below and in S1 Appendix. In Table 2, we summarize approaches for measuring care cascade outcomes and suggest process indicators for each cascade gap that may reveal deficiencies in quality of care. Data for process indicators could be collected concurrently with cohort-based studies aiming to measure care cascade outcomes.

**Table 2:** Recommended outcome and process indicators for a care cascade for active TB.

Cascade stage	Outcome indicators for cascade steps (useful for monitoring program outcomes)	Methods or required data for outcome indicators	Process indicators for cascade gaps^a^ (useful for understanding quality of care)	Methods or required data for process indicators
**Stage 1: Reaching health facilities and accessing a TB test**	**Step 1: Number of individuals with incident or prevalent TB in the population**		**Gap 1: Number of individuals with TB who did not reach health facilities and access a TB diagnostic test**^**b**^	
	Number of individuals with prevalent active TB in a population for each form of TB	Population-based TB prevalence survey, including drug-susceptibility testing and prior TB treatment history for diagnosed patients	Distance to nearest TB health facility as a surrogate measure of the proportion of individuals without access to TB services^c^	Questions asked to TB patients diagnosed in population-based prevalence surveys
	Annual number of individuals with incident active TB in a population for each form of TB	Modeling methods may facilitate estimation of incidence from active TB prevalence, surveys of the annual risk of TB infection, government case notifications, TB drug sales, or other data	Proportion who have not sought medical care	Questions asked to TB patients diagnosed in population-based prevalence surveys
			Time delays in care seeking^d^	In-depth interviews with individuals starting TB treatment at health facilities^d^
			Number of individuals who died of TB without having received TB care	Population-based verbal autopsy surveys, including in-depth interviews with families of individuals who died of probable TB
**Stage 2: Diagnosis**	**Step 2: Number of individuals with TB who reached health facilities and accessed a TB diagnostic test**^**b**^		**Gap 2: Number of individuals with TB who accessed a TB diagnostic test**^**b**^ **but did not get successfully diagnosed**	
	Number of individuals with smear-positive TB who accessed TB tests	Extrapolation from the proportion of patients who did not submit a second sputum sample (S1 Appendix)	Proportion of individuals with suspected TB who did not undergo any sputum testing	Audit of patient records at TB diagnostic facilities
	Number of individuals with Xpert-positive TB who accessed TB tests	Number evaluated equals the number diagnosed		
	Number of individuals with smear- or Xpert-negative TB who accessed TB tests or who had initiation of appropriate workup	Estimation based on the sensitivity of sputum microscopy or Xpert MTB/RIF in a given setting (S1 Appendix)	Proportion of individuals with suspected TB with negative sputum microscopy or Xpert test results who do not receive a medical diagnosis	Audit of patient records at TB diagnostic facilities
	Number of individuals with extrapulmonary TB who had initiation of appropriate workup	Estimation based on the anticipated rate of underdiagnosis of extrapulmonary TB in a given setting (S1 Appendix)		
	Number of individuals with MDR or RR TB who accessed TB tests	Extrapolation from culture-based studies estimating the proportion of MDR/RR TB among new and previously treated patients in a given setting (S1 Appendix)		
			Health system–related delays in diagnosis^d^	In-depth interviews with patients starting TB treatment^d^
**Stage 3: Linkage to treatment**	**Step 3: Number of individuals diagnosed with TB**^**e**^		**Gap 3: Number of individuals diagnosed with TB who did not get registered in treatment**	
	Number of individuals with smear- or Xpert-positive (i.e., bacteriologically diagnosed) TB who were successfully diagnosed	Data on bacteriologically diagnosed pulmonary TB patients is usually efficiently captured in patient registers at diagnostic facilities	Proportion of patients lost prior to referral from a TB diagnostic facility to a treatment facility	Audit of diagnostic and referral registers at TB diagnostic facilities
	Number of individuals with smear-negative, Xpert-negative, or extrapulmonary TB who were successfully diagnosed	These patients have more prolonged diagnostic workups and may be listed in separate registers from bacteriologically diagnosed pulmonary TB patients, such as registers used to refer patients to treatment sites	Proportion of patients lost after referral from the TB diagnostic facility to a treatment facility	Audit of referral registers at TB diagnostic facilities and registers at treatment facilities
	Number of individuals with MDR TB or RR TB who were successfully diagnosed as having drug-resistant TB	These patients can be identified through lab registers recording drug-susceptibility test results. Otherwise, they may be misclassified as drug-susceptible TB patients	Delays in treatment initiation^d^	In-depth interviews with patients starting TB treatment^d^
**Stage 4: Retention in treatment**	**Step 4: Number of individuals registered in TB treatment**^**e**^		**Gap 4: Number of individuals who did not complete TB treatment (due to treatment failure, loss to follow-up, or death)**	
	Number of individuals registered (or notified) in TB treatment	TB treatment records or electronic registers	Proportion of patients who experience treatment failure, die, or are lost to follow-up in the intensive phase of therapy	TB treatment records
			Proportion of patients who experience treatment failure, die, or are lost to follow-up in the continuation phase of therapy	TB treatment records
			Proportion of expected doses of TB medication actually taken during the treatment course (measure of the quality of medication adherence) [54]	TB treatment records
**Stage 5: Post-treatment survival**	**Step 5: Number of individuals who completed TB treatment**^**e**^		**Gap 5: Number of individuals who experienced post-treatment TB recurrence or death**	
	Number of patients who complete TB therapy	TB treatment records or electronic registers	Proportion of patients who experience TB recurrence or death within 1 year of treatment completion	Cohort studies involving close follow-up of patients every few months after treatment, with careful workup of new pulmonary symptoms, ideally with mycobacterial culture
			Proportion of patients with post-TB lung disease, including obstructive disease, restrictive/fibrotic disease, and pulmonary hypertension	Routine post-treatment follow-up of patients with spirometry and other measures of pulmonary function
**Stage 6: Achieving durable cure**	**Step 6: Number of individuals who achieve 1-year recurrence-free survival**^**e**^			
	Number of patients who survive for 1 year after completing TB treatment without disease recurrence	Cohort studies involving close follow-up of patients every few months after treatment up to 12 months, with careful workup of any new pulmonary symptoms, ideally with mycobacterial culture		

^a^ Gaps can be estimated as the difference between two steps (i.e., Gap 1 = Step 1 − Step 2). The process indicators described in the table will further inform reasons for each gap.

^b^ “Accessed a TB diagnostic test” refers to individuals with TB who either accessed an appropriate bacteriological test for TB or who had initiation of appropriate workup (for extrapulmonary or pulmonary TB patients who might be diagnosed empirically).

^c^ Distance of a patient’s home from the nearest health facility is only one aspect of access to care; other factors include economic and social barriers, though these may be harder to measure routinely.

^d^ Single in-depth interviews with TB patients at the time of treatment initiation can be used to capture information on delays in care seeking, diagnosis, and treatment initiation.

^e^ Steps 3, 4, 5, and 6 are best estimated by following a single patient cohort, starting with diagnosed TB patients identified in Step 3 (i.e., a cohort-based or denominator–denominator linked approach).

**Abbreviations:** MDR, multidrug-resistant; RR, rifampin-resistant; TB, tuberculosis.

#### Stage 1: Reaching health facilities and accessing a TB test

Estimating the number of individuals with active TB in a population (Step 1) is valuable for national-level cascades because the number of individuals with TB who do not access a TB test (Gap 1) may be a large gap and may contribute considerably to TB transmission [10]. The annual number of individuals with incident TB is the ideal metric for Step 1 because most programs report subsequent outcomes, such as the number of individuals who complete treatment, on a yearly basis.

For most countries, incidence and prevalence estimates are routinely reported by WHO and are informed by country experts [24]. Alternative estimates are available from the Institute for Health Metrics and Evaluation (IHME) [55,56]. However, WHO and IHME incidence estimates are partly extrapolated from notification data, which may have inaccuracies, especially where the private sector delivers a large proportion of TB care [26,57]. When possible, we suggest validating WHO or IHME estimates against independent sources of information on TB burden, such as private sector TB drug sales [26]. Mathematical models, incorporating data from population-based surveys of active or latent TB prevalence and mortality [27], may also be informative. Moreover, population-based prevalence surveys provide objective data on the number of individuals with active TB in the population, which can be used for longitudinal monitoring [58]. Prevalence surveys may also provide information on Gap 1 process indicators (Tables 2 and 3), which can be used to monitor the population’s care-seeking behavior and the impact of TB public education programs on modifying this behavior.

**Table 3:** Survey data that can be collected during active TB prevalence surveys, in addition to standard diagnostic tests, to facilitate estimation of care cascade outcome and process indicators.

Survey questions for individuals diagnosed with active TB in a prevalence survey	Benefit for understanding care cascade outcomes and process indicators
History of prior TB treatment	Estimation of the proportion of individuals with active TB who have a prior TB treatment history in the population
Nearest government facility with TB services	Estimation of proportion of individuals with active TB who may not have adequate access to TB services
Whether the patient has sought care for TB symptoms	Indirect evidence of the proportion of incident cases seeking care and of the delay before doing so
If care was sought, whether the patient was screened with a sputum test or chest X-ray	Indirect evidence of the proportion of incident cases with access to TB diagnostic tests and a measure of quality of care
Duration of TB symptoms	May help to model annual incidence from point prevalence; indirect evidence of delays in seeking care

For Gap 1, individuals who die without accessing TB care are particularly concerning. Achieving accurate estimates of these individuals is challenging, given limitations in the accuracy of vital registration systems and medical certification of causes of death in many countries. Verbal autopsy may help refine TB mortality estimates in such settings [59].

#### Stage 2: Diagnosis

We define Stage 2 starting from when individuals with pulmonary TB reach a health facility and access TB tests (e.g., sputum microscopy, Xpert MTB/RIF) or when appropriate workup is initiated by a healthcare provider for individuals with extrapulmonary or pulmonary TB who might be diagnosed empirically. While estimating Stage 2 requires different methods for each form of TB, it provides valuable insights on gaps in care. For example, in the Indian and South African TB care cascades, about 310,000 (16% of those tested) and 69,000 (14% of those tested), respectively, were not successfully diagnosed or never received their diagnosis [10,11]. Estimating Gap 2 is especially valuable for smear-negative, Xpert-negative, and drug-resistant TB, which are more difficult to diagnose. This gap may reveal patient losses from use of suboptimal diagnostic tests (e.g., sputum microscopy) or from poor adherence to algorithms for empirical diagnosis.

Individuals with smear-positive TB evaluated with sputum microscopy are, by definition, likely to be diagnosed [60]. A small proportion may be missed if they do not submit a second sputum sample (S1 Appendix), especially in locations where same-day microscopy has not been implemented [61]. In settings using Xpert, because a single sputum sample is usually submitted, the number of individuals with Xpert-positive TB who access the test (Step 2) can be assumed to be the same as the number who get diagnosed with Xpert-positive TB (Step 3).

In settings without more advanced diagnostic tests, individuals with smear-negative TB are diagnosed empirically. Most individuals who have negative sputum smears have conditions other than TB (e.g., bacterial pneumonia), making it challenging to estimate the number of true smear-negative TB patients evaluated at diagnostic facilities. Because the number of individuals with smear-positive TB in Step 2 can be more reliably estimated, the estimated ratio of individuals with smear-negative to smear-positive TB in a setting (a reflection of the sensitivity of sputum microscopy compared to a gold standard of culture) can be used to roughly estimate the number of true smear-negative TB patients who get evaluated at diagnostic facilities (S1 Appendix). Estimates of this ratio may be more relevant if based on high-quality local studies of the sensitivity of sputum microscopy in programmatic conditions [10]. In settings using Xpert MTB/RIF as the primary test, a similar ratio method based on estimates of Xpert’s sensitivity can be used to estimate Step 2 for individuals with Xpert-negative TB (S1 Appendix).

Estimating the number of true extrapulmonary TB patients who access appropriate workup is also challenging because clinical presentation and sensitivity of diagnostic tests vary depending on the site of disease. Studies that identify individuals with possible extrapulmonary TB who present to diagnostic facilities and follow them to determine the number who complete appropriate workup and get diagnosed may inform Step 2 and Gap 2 estimates. The number of MDR (or rifampin-resistant) TB patients reaching health facilities and accessing a TB test (Step 2) can be estimated using MDR TB rates in new and previously treated patients, which are available for most countries from WHO [23] or national MDR TB prevalence surveys [62] (S1 Appendix). Finally, estimating Stage 2 for children can be particularly challenging because of the low sensitivity of diagnostic tests in this population [63,64] (S1 Appendix).

#### Stage 3: Linkage to treatment

PTLFU—loss of diagnosed patients prior to treatment registration—is a major point of attrition in TB programs [10,11,65]. Most studies have examined this gap for smear-positive [10,65] or drug-resistant TB patients [66–70]. Few have examined this gap for smear-negative [71,72], Xpert-negative, or extrapulmonary TB patients. Future care cascade analyses should estimate this gap for all forms of TB.

To measure PTLFU, many studies identify newly diagnosed TB patients in registers at diagnostic facilities and prospectively track them to see if they get registered at treatment centers, an approach which can also facilitate cohort-based estimates for remaining cascade stages (Table 2). While we agree with this approach, it can be challenging for a few reasons. First, in some settings, TB treatment initiation and official registration (or notification) do not happen concurrently. Patients may be lost to follow-up after starting therapy but before official treatment registration [32]. Second, patients may get diagnosed in one location (e.g., a city) but start treatment elsewhere (e.g., a rural area), making follow-up difficult, especially since unique identification numbers are uncommon in many countries [31,32,73]. Third, missing or illegible contact information often makes patient tracking difficult, especially in settings using paper records [31–33,73,74].

Capturing patient information in electronic registration systems at diagnosis and treatment initiation may improve estimation of PTLFU [66]. South Africa has introduced unique patient identification numbers along with a national electronic notification system to ensure patients attending different facilities are not counted multiple times. India is rolling out a similar system. Such systems may facilitate patient tracking across large geographic areas. Officially registering (i.e., notifying) patients at the time of diagnosis, as India is trying to do, may also improve estimation of PTLFU.

Finally, interviewing patients at the time of treatment registration allows assessment of delays in care seeking, diagnosis, and treatment initiation, which are helpful process indicators (Table 2) [42,75]. Some interventions may impact PTLFU and time delays differently. For example, a South African study found that use of Xpert reduced treatment delays for rifampin-resistant TB patients without reducing PTLFU [66].

#### Stage 4: Retention in treatment

Most national TB programs routinely report data on patients registered in treatment (Step 4) and who do not complete therapy (Gap 4), based on the WHO guidelines [76]. Suboptimal Gap 4 outcomes consist of patients who are lost to follow-up, experience treatment failure (i.e., positive sputum smear or culture despite therapy), or die while on treatment [76].

While estimating Stage 4 using aggregate numbers from TB programs may be helpful, we recommend using prospective patient-tracking approaches that allow for rigorous cohort-based care cascade estimates. For this approach, patients diagnosed with TB in Step 3 can be followed through Step 4 (treatment registration) and Step 5 (treatment completion) using clinical records (Table 2). This approach also allows elucidation of the time during treatment when most poor outcomes occur (e.g., intensive or continuation phase). Digital adherence technologies—including digital pillboxes and cell phone–based strategies—may also facilitate more accurate estimation of Stage 4 and timing of patient losses [54,77].

#### Stages 5 and 6: Post-treatment survival and achieving durable cure

Step 5 (treatment completion) can be assessed using treatment cards or registers in most national TB programs [76]. However, estimating Step 6 (1-year recurrence-free survival) requires following patients after treatment completion. Post-treatment follow-up is not routine in most programs, though some national guidelines recommend such monitoring [15,78,79]. Studies show high rates of post-treatment disease recurrence and death under programmatic conditions, highlighting the importance of evaluating these longer-term outcomes [25,80–83].

Post-treatment disease recurrence is an indicator of quality of care, since recurrence may result from poor medication adherence during therapy [80,84] or undiagnosed drug resistance [25,85]. In settings where HIV coinfection is common, disease recurrence is often due to exogenous reinfection with a new TB strain [86,87]. One-year TB recurrence-free survival may be a less useful outcome for the cascade in such settings, although high recurrence rates in these settings may indicate need for transmission control interventions. We recommend 12 months of post-treatment follow-up because most cases of TB relapse (91%) occur in this time period, based on a meta-analysis of clinical trials [88].

To achieve accurate Gap 5 and Step 6 estimates, we recommend a cohort-based approach with prospective follow-up of patients who complete treatment because retrospective follow-up of patients who finish treatment may be compromised by higher loss to follow-up. In addition, Gap 5 can most accurately be estimated by collecting sputum samples for mycobacterial culture from symptomatic patients (for those who had pulmonary TB) or repeated clinical evaluation (for those who had extrapulmonary TB), which is not possible to do retrospectively. Patients who complete TB treatment should ideally be regularly reevaluated (e.g., every 3 months), for at least 1 year [36,80].

## Discussion

The care cascade represents a valuable and feasible approach for monitoring TB programs [10]. Unique challenges involved in constructing a TB care cascade include difficulties in estimating the number of individuals with active TB in the population, challenges in estimating the diagnostic gap (Gap 2) due to the suboptimal sensitivity of common diagnostic tests, and heterogeneity in approaches for estimating cascade stages for different forms of TB. In addition, the case-finding gap (Gap 1) includes individuals with TB who do not access TB tests for various reasons, including not having access to health facilities, not seeking care, and not being referred for TB testing after reaching a healthcare provider. Understanding which barrier contributes most to Gap 1 is an important undertaking that we have not covered in this manuscript. Some challenges involved in estimating the care cascade are not unique to TB—for example, use of written records and lack of unique identification numbers, which makes tracking patients across stages more difficult. Additionally, it is not easy to account for patients managed in the private sector in some countries, without conducting primary data collection.

Despite these challenges, key cascade stages can be evaluated in most settings. While robust estimates of the number of individuals with active TB in the population may not always be available, cohort studies can be implemented in most settings starting from Stage 2 or 3 to estimate subsequent stages. Even without estimates of the number of individuals with active TB in the population, these research approaches can provide valuable insights for strengthening health systems by identifying gaps with the largest patient losses.

There are limitations in the scope of what the care cascade model measures. For example, delays in care seeking, diagnosis, and treatment initiation may not be adequately captured; however, as described above, the care cascade also provides a framework for understanding how patients traverse stages in care, into which other process indicators can be embedded. If cohort-based approaches are used to measure the care cascade, data on some of these process indicators can be collected concurrently to gain additional insights into quality of care.

Ideally, care cascade estimates would rely on robust survey data and longitudinal monitoring by health systems, including nationally representative TB prevalence and mortality data, electronic medical records for capturing notification and outcomes of private sector TB patients, and routine post-treatment follow-up to estimate TB recurrence. Countries currently have variable availability of these data and infrastructure.

**Fig 3:**
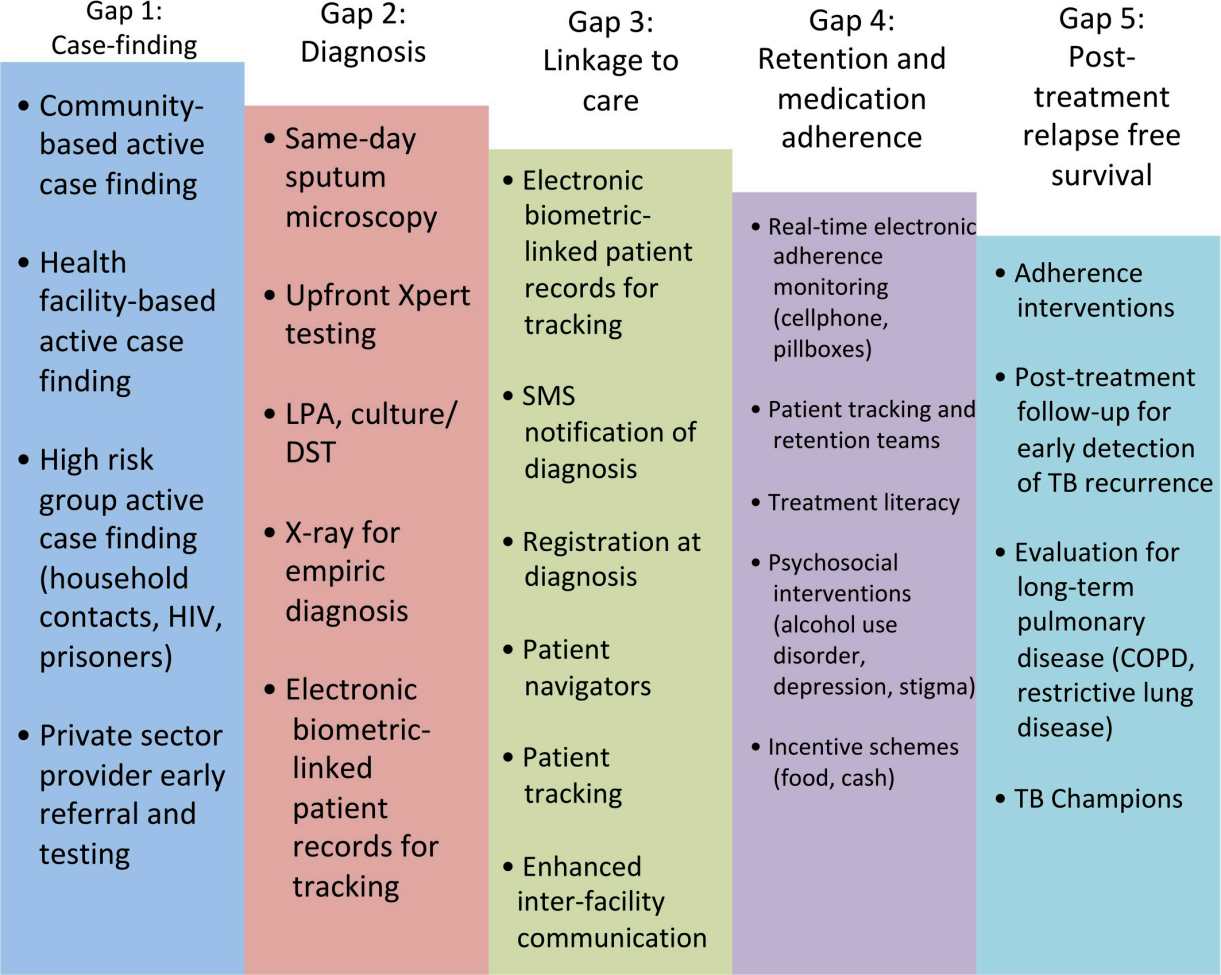
An example of how potential interventions can be mapped onto different gaps to address patient losses in the TB care cascade. Different interventions might be chosen based on the setting. We do not cover the evidence basis for these interventions here. TB Champions refers to individuals who have survived TB who serve as advocates to increase awareness and support for patients with active TB who are in treatment or who have completed treatment [89]. COPD, chronic obstructive pulmonary disease; DST, drug susceptibility testing; LPA, line probe assay; SMS, short messaging service; TB, tuberculosis.

Patient outcomes may be improved by implementing interventions addressing the most concerning gaps, which may be related to case finding, diagnostic workup, linkage to treatment, retention in care, or medication adherence (to reduce TB recurrence) (Fig 3). Patient mobility (e.g., urban–rural travel) is a barrier for ensuring linkage to, and retention in, care in many settings [31]. Written records often require healthcare workers to track patients through different paper registers for diagnosis, drug susceptibility testing, treatment initiation, and treatment monitoring, which may contribute to diagnostic and treatment delays.

Robust electronic systems with unique identification numbers for tracking patients, linking them to care, and monitoring medication adherence in real time have the potential to improve gaps in the care cascade [54,90]. Once patients are started on treatment, a holistic management approach, including provision of economic incentives and enablers, nutritional support, and care for comorbidities (e.g., substance use, depression), may also improve outcomes [91].

Although important information can be obtained from routine programmatic data, dedicated cohort studies will yield the most accurate care cascade estimates, especially for stages such as recurrence-free survival, for which programs may not routinely collect data. If representative sampling is used, multisite cohort studies can produce accurate national-level care cascade estimates that could be used for longitudinal monitoring of outcomes.

## Conclusion

The care cascade has the potential to improve program monitoring and to inform targeting of interventions to improve case finding, diagnosis, linkage to treatment, retention in care, and recurrence-free survival for TB patients. Combined with other approaches, such as patient pathways analyses, the care cascade can provide critical information on quality of care to national TB programs [12]. The model may refine estimates for the STOP TB Partnership’s 90-(90)-90 global targets, which include getting 90% of people with active TB on appropriate therapy, reaching at least 90% of key high-risk or underserved populations as part of this approach, and ensuring that 90% of those patients achieve treatment success by 2025 at the latest. By providing a systematic approach to evaluating care delivery, followed by corrective interventions, the care cascade may serve as an important tool for achieving the ambitious goal of reducing TB incidence by 90% by 2035, as envisioned by the End TB strategy [92].

## Supporting information

S1 Appendix Constructing a tuberculosis cascade of care: a “how to” guide. (PDF)

## Abbreviations

COPD: chronic obstructive pulmonary disease
DST: drug susceptibility testing
IHME: Institute for Health Metrics and Evaluation
LPA: line probe assay
MDR TB: multidrug-resistant tuberculosis
PCR: polymerase chain reaction
PTLFU: pretreatment loss to follow-up
TB: tuberculosis
UNAIDS: Joint United Nations Programme on HIV/AIDS
WHO: World Health Organization

## References

[1] World Health Organization. Global tuberculosis report. Geneva: WHO, 2015 Contract No.: WHO/HTM/TB/2015.22.

[2] World Health Organization. Global strategy and targets for tuberculosis prevention, care, and control after 2015. Geneva: WHO, 2013 Contract No.: EB134/12.

[3] Nosyk B, Montaner JS, Colley G, Lima VD, Chan K, Heath K, et al The cascade of HIV care in British Columbia, Canada, 1996–2011: a population-based retrospective cohort study. Lancet Infect Dis. 2014;14(1):40–9. 10.1016/S1473-3099(13)70254-8 . 24076277 PMC4017913

[4] Mehta SH, Lucas GM, Solomon S, Srikrishnan AK, McFall A, Nandagopal P, et al HIV Care Cascade Among Hard To Reach Populations in India: Need To Expand HIV Counseling and Testing. Top Antivir Med. 2014;22(e-1):565.

[5] Haber N, Tanser F, Bor J, Naidu K, Mutevedzi T, Herbst K, et al From HIV infection to therapeutic response: a population-based longitudinal HIV cascade-of-care study in KwaZulu-Natal, South Africa. Lancet HIV. 2017;4(5):E223–E30. 10.1016/S2352-3018(16)30224-7 . 28153470 PMC5964602

[6] Ali MK, Bullard KM, Gregg EW, Del Rio C. A cascade of care for diabetes in the United States: visualizing the gaps. Ann Intern Med. 2014;161(10):681–9. 10.7326/M14-0019 . 25402511

[7] Yehia BR, Schranz AJ, Umscheid CA, Lo Re V. The treatment cascade for chronic hepatitis C virus infection in the United States: a systematic review and meta-analysis. PLoS ONE. 2014;9(7):e101554 10.1371/journal.pone.0101554 . 24988388 PMC4079454

[8] UNAIDS. 90-90-90: An ambitious treatment target to help end the AIDS epidemic. Geneva: Joint United Nations Programme on HIV/AIDS, 2014 Contract No.: JC2684.

[9] Levi J, Raymond A, Pozniak A, Vernazza P, Kohler P, Hill A. Can the UNAIDS 90-90-90 target be achieved? A systematic analysis of national HIV treatment cascades. BMJ Glob Health. 2016;1:e000010 10.1136/bmjgh-2015-000010 28588933 PMC5321333

[10] Subbaraman R, Nathavitharana RR, Satyanarayana S, Pai M, Thomas BE, Chadha VK, et al The Tuberculosis Cascade of Care in India's Public Sector: A Systematic Review and Meta-analysis. PLoS Med. 2016;13(10):e1002149 10.1371/journal.pmed.1002149 . 27780217 PMC5079571

[11] Naidoo P, Theron G, Rangaka MX, Chihota VN, Vaughan L, Brey ZO, et al The South African Tuberculosis Care Cascade: Estimated Losses and Methodological Challenges. J Infect Dis. 2017;216(suppl_7):S702–S13. 10.1093/infdis/jix335 . 29117342 PMC5853316

[12] Hanson CL, Osberg M, Brown J, Durham G, Chin DP. Conducting Patient-Pathway Analysis to Inform Programming of Tuberculosis Services: Methods. J Infect Dis. 2017;216(suppl_7):S679–S85. 10.1093/infdis/jix387 . 29117350 PMC5853893

[13] Wells WA. Onions and prevalence surveys: how to analyze and quantify tuberculosis case-finding gaps. Int J Tuberc Lung Dis. 2017;21(11):1101–13. 10.5588/ijtld.17.0271 . 29037290

[14] Reid AJ, Goosby E. Lessons learned from the HIV care cascade can help End TB. Int J Tuberc Lung Dis. 2017;21(3):245–6. 10.5588/ijtld.17.0027 28225330

[15] Central TB Division. India's National Strategic Plan for TB Elimination 2017–2025. New Delhi: Ministry of Health and Family Welfare, 2017.

[16] South African National AIDS Council. South African National Strategic Plan on HIV, TB and STIs 2017–2022 (Draft 2). Pretoria: South African National AIDS Council, 2017.

[17] Drain PK, Bajema KL, Dowdy D, Dheda K, Naidoo K, Schumacher SG, et al Incipient and Subclinical: a Clinical Review of Early Stages and Progression of Infection. Clin Microbiol Rev. 2018;31(4):e00021–18. 10.1128/cmr.00021-18 . 30021818 PMC6148193

[18] Houben RM, Dodd PJ. The Global Burden of Latent Tuberculosis Infection: A Re-estimation Using Mathematical Modelling. PLoS Med. 2016;13(10):e1002152 10.1371/journal.pmed.1002152 . 27780211 PMC5079585

[19] Alsdurf H, Hill PC, Matteelli A, Getahun H, Menzies D. The cascade of care in diagnosis and treatment of latent tuberculosis infection: a systematic review and meta-analysis. Lancet Infect Dis. 2016;16(11):1269–78. 10.1016/S1473-3099(16)30216-X . 27522233

[20] Mwangwa F, Chamie G, Kwarisiima D, Ayieko J, Owaraganise A, Ruel TD, et al Gaps in the Child Tuberculosis Care Cascade in 32 Rural Communities in Uganda and Kenya. J Clin Tuberc Other Mycobact Dis. 2017;9:24–9. 10.1016/j.jctube.2017.10.003 . 29291251 PMC5743212

[21] Hanrahan CF, Van Rie A. A proposed novel framework for monitoring and evaluation of the cascade of HIV-associated TB care at the health facility level. J Int AIDS Soc. 2017;20(1):21375 10.7448/IAS.20.01.21375 . 28440604 PMC5515049

[22] Armstrong-Hough M, Turimumahoro P, Meyer AJ, Ochom E, Babirye D, Ayakaka I, et al Drop-out from the tuberculosis contact investigation cascade in a routine public health setting in urban Uganda: A prospective, multi-center study. PLoS ONE. 2017;12(11):e0187145 10.1371/journal.pone.0187145 . 29108007 PMC5673209

[23] World Health Organization. Global tuberculosis report. Geneva: World Health Organization, 2016 Contract No.: WHO/HTM/TB/2016.13.

[24] World Health Organization. Global tuberculosis report. Geneva: World Health Organization, 2017 Contract No.: WHO/HTM/TB/2017.23.

[25] Cox H, Kebede Y, Allamuratova S, Ismailov G, Davletmuratova Z, Byrnes G, et al Tuberculosis recurrence and mortality after successful treatment: Impact of drug resistance. PLoS Med. 2006;3(10):1836–43. 10.1371/journal.pmed.0030384 . 17020405 PMC1584414

[26] Arinaminpathy N, Batra D, Khaparde S, Vualnam T, Maheshwari N, Sharma L, et al The number of privately treated tuberculosis cases in India: an estimation from drug sales data. Lancet Infect Dis. 2016;16:1255–60. 10.1016/S1473-3099(16)30259-6 . 27568356 PMC5067370

[27] Pandey S, Chadha VK, Laxminarayan R, Arinaminpathy N. Estimating tuberculosis incidence from primary survey data: a mathematical modeling approach. Int J Tuberc Lung Dis. 2017;21(4):366–74. 10.5588/ijtld.16.0182 . 28284250 PMC5347365

[28] Albert H, Nathavitharana RR, Isaacs C, Pai M, Denkinger CM, Boehme CC. Development, roll-out and impact of Xpert MTB/RIF for tuberculosis: what lessons have we learnt and how can we do better? Eur Respir J. 2016;48(2):516–25. 10.1183/13993003.00543-2016 . 27418550 PMC4967565

[29] Pai M, Schito M. Tuberculosis diagnostics in 2015: landscape, priorities, needs, and prospects. J Infect Dis. 2015;211 Suppl 2:S21–8. 10.1093/infdis/jiu803 . 25765103 PMC4366576

[30] Salje H, Andrews JR, Deo S, Satyanarayana S, Sun AY, Pai M, et al The importance of implementation strategy in scaling up Xpert MTB/RIF for diagnosis of tuberculosis in the Indian health-care system: a transmission model. PLoS Med. 2014;11(7):e1001674 10.1371/journal.pmed.1001674 . 25025235 PMC4098913

[31] Subbaraman R, Thomas BE, Sellappan S, Suresh C, Jayabal L, Lincy S, et al Tuberculosis patients in an Indian mega-city: Where do they live and where are they diagnosed? PLoS ONE. 2017;12(8):e0183240 10.1371/journal.pone.0183240 . 28813536 PMC5557603

[32] Thomas BE, Subbaraman R, Sellappan S, Suresh C, Lavanya J, Lincy S, et al Pretreatment loss to follow-up of tuberculosis patients in Chennai, India: a cohort study with implications for health systems strengthening. BMC Infect Dis. 2018;18(1):142 10.1186/s12879-018-3039-3 29587651 PMC5872574

[33] Sai Babu B, Satyanarayana AV, Venkateshwaralu G, Ramakrishna U, Vikram P, Sahu S, et al Initial default among diagnosed sputum smear-positive pulmonary tuberculosis patients in Andhra Pradesh, India. Int J Tuberc Lung Dis. 2008;12(9):1055–8. . 18713504

[34] Jha UM, Satyanarayana S, Dewan PK, Chadha S, Wares F, Sahu S, et al Risk factors for treatment default among re-treatment tuberculosis patients in India, 2006. PLoS ONE. 2010;5(1):e8873 10.1371/journal.pone.0008873 . 20111727 PMC2810342

[35] Chadha VK, Praseeja P, Hemanthkumar NK, Shivshankara BA, Sharada MA, Nagendra N, et al Implementation efficiency of a diagnostic algorithm in sputum smear-negative presumptive tuberculosis patients. Int J Tuberc Lung Dis. 2014;18(10):1237–42. 10.5588/ijtld.14.0218 . 25216839

[36] Velayutham B, Chadha VK, Singla N, Narang P, Gangadhar Rao V, Nair S, et al Recurrence of tuberculosis among newly diagnosed sputum positive pulmonary tuberculosis patients treated under the Revised National Tuberculosis Control Programme, India: A multi-centric prospective study. PLoS ONE. 2018;13(7):e0200150 10.1371/journal.pone.0200150 . 29979738 PMC6034867

[37] Ahmed S, Autrey J, Katz IT, Fox MP, Rosen S, Onoya D, et al Why do people living with HIV not initiate treatment? A systematic review of qualitative evidence from low- and middle-income countries. Soc Sci Med. 2018;213:72–84. 10.1016/j.socscimed.2018.05.048 . 30059900 PMC6813776

[38] Escudero DJ, Lurie MN, Mayer KH, King M, Galea S, Friedman SR, et al The risk of HIV transmission at each step of the HIV care continuum among people who inject drugs: a modeling study. BMC Public Health. 2017;17(1):614 10.1186/s12889-017-4528-9 . 28738861 PMC5525346

[39] Risher K, Mayer KH, Beyrer C. HIV treatment cascade in MSM, people who inject drugs, and sex workers. Curr Opin HIV AIDS. 2015;10(6):420–9. 10.1097/COH.0000000000000200 . 26352393 PMC4880053

[40] Zanoni BC, Mayer KH. The adolescent and young adult HIV cascade of care in the United States: exaggerated health disparities. AIDS Patient Care STDS. 2014;28(3):128–35. 10.1089/apc.2013.0345 . 24601734 PMC3948479

[41] Haber N, Pillay D, Porter K, Barnighausen T. Constructing the cascade of HIV care: methods for measurement. Curr Opin HIV AIDS. 2016;11(1):102–8. 10.1097/COH.0000000000000212 . 26545266

[42] Sreeramareddy CT, Qin ZZ, Satyanarayana S, Subbaraman R, Pai M. Delays in diagnosis and treatment of pulmonary tuberculosis in India: a systematic review. Int J Tuberc Lung Dis. 2014;18(3):255–66. 10.5588/ijtld.13.0585 . 24670558 PMC4070850

[43] Justman JE, Mugurungi O, El-Sadr WM. HIV Population Surveys—Bringing Precision to the Global Response. N Engl J Med. 2018;378(20):1859–61. 10.1056/NEJMp1801934 . 29768142

[44] Steingart KR, Schiller I, Horne DJ, Pai M, Boehme CC, Dendukuri N. Xpert(R) MTB/RIF assay for pulmonary tuberculosis and rifampicin resistance in adults. Cochrane Database Syst Rev. 2014;1:Cd009593 10.1002/14651858.CD009593.pub3 . 24448973 PMC4470349

[45] Davis JL, Cattamanchi A, Cuevas LE, Hopewell PC, Steingart KR. Diagnostic accuracy of same-day microscopy versus standard microscopy for pulmonary tuberculosis: a systematic review and meta-analysis. Lancet Infect Dis. 2013;13(2):147–54. 10.1016/S1473-3099(12)70232-3 . 23099183 PMC3836432

[46] Satyanarayana S, Nair SA, Chadha SS, Shivashankar R, Sharma G, Yadav S, et al From where are tuberculosis patients accessing treatment in India? Results from a cross-sectional community based survey of 30 districts. PLoS ONE. 2011;6(9):e24160 10.1371/journal.pone.0024160 . 21912669 PMC3166304

[47] Surya A, Setyaningsih B, Suryani Nasution H, Gita Parwati C, Yuzwar YE, Osberg M, et al Quality Tuberculosis Care in Indonesia: Using Patient Pathway Analysis to Optimize Public-Private Collaboration. J Infect Dis. 2017;216(suppl_7):S724–S32. 10.1093/infdis/jix379 . 29117347 PMC5853837

[48] Fatima R, Harris RJ, Enarson DA, Hinderaker SG, Qadeer E, Ali K, et al Estimating tuberculosis burden and case detection in Pakistan. Int J Tuberc Lung Dis. 2014;18(1):55–60. 10.5588/ijtld.13.0198 . 24365553

[49] Khan AJ, Khowaja S, Khan FS, Qazi F, Lotia I, Habib A, et al Engaging the private sector to increase tuberculosis case detection: an impact evaluation study. Lancet Infect Dis. 2012;12(8):608–16. 10.1016/S1473-3099(12)70116-0 . 22704778

[50] Hanson C, Osberg M, Brown J, Durham G, Chin DP. Finding the Missing Patients With Tuberculosis: Lessons Learned From Patient-Pathway Analyses in 5 Countries. J Infect Dis. 2017;216(suppl_7):S686–S95. 10.1093/infdis/jix388 . 29117351 PMC5853970

[51] McDowell A, Pai M. Treatment as diagnosis and diagnosis as treatment: empirical management of presumptive tuberculosis in India. Int J Tuberc Lung Dis. 2016;20(4):536–43. 10.5588/ijtld.15.0562 . 26970165

[52] Das J, Kwan A, Daniels B, Satyanarayana S, Subbaraman R, Bergkvist S, et al Use of standardised patients to assess quality of tuberculosis care: a pilot, cross-sectional study. Lancet Infect Dis. 2015;15(11):1305–13. 10.1016/S1473-3099(15)00077-8 . 26268690 PMC4633317

[53] Kwan A, Daniels B, Saria V, Satyanarayana S, Subbaraman R, McDowell A, et al Variations in the quality of tuberculosis care in urban India: A cross-sectional, standardized patient study in two cities. PLoS Med. 2018;15(9):e1002653 10.1371/journal.pmed.1002653 . 30252849 PMC6155454

[54] Subbaraman R, de Mondesert L, Musiimenta A, Pai M, Mayer KH, Thomas BE, et al Digital adherence technologies for the management of tuberculosis therapy: mapping the landscape and research priorities. BMJ Glob Health. 2018;3:e001018 10.1136/bmjgh-2018-001018 30364330 PMC6195152

[55] Garcia-Basteiro AL, Brew J, Williams B, Borgdorff M, Cobelens F. What is the true tuberculosis mortality burden? Differences in estimates by the World Health Organization and the Global Burden of Disease study. Int J Epidemiol. 2018;46(5):1549–60. 10.1093/ije/dyy144 . 30010785

[56] Kyu HH, Maddison ER, Henry NJ, Ledesma JR, Wiens KE, Reiner R Jr., et al Global, regional, and national burden of tuberculosis, 1990–2016: results from the Global Burden of Diseases, Injuries, and Risk Factors 2016 Study. Lancet. 2018;18(12):1329–49. 10.1016/S1473-3099(18)30625-X 30507459 PMC6250050

[57] Cowling K, Dandona R, Dandona L. Improving the estimation of the tuberculosis burden in India. Bull World Health Organ. 2014;92(11):817–25. 10.2471/BLT.13.129775 . 25378743 PMC4221760

[58] Wang L, Zhang H, Ruan Y, Chin DP, Xia Y, Cheng S, et al Tuberculosis prevalence in China, 1990–2010; a longitudinal analysis of national survey data. Lancet. 2014;383(9934):2057–64. 10.1016/S0140-6736(13)62639-2 . 24650955

[59] Jha P, Gajalakshmi V, Gupta PC, Kumar R, Mony P, Dhingra N, et al Prospective study of one million deaths in India: rationale, design, and validation results. PLoS Med. 2006;3(2):e18 10.1371/journal.pmed.0030018 . 16354108 PMC1316066

[60] Mase SR, Ramsay A, Ng V, Henry M, Hopewell PC, Cunningham J, et al Yield of serial sputum specimen examinations in the diagnosis of pulmonary tuberculosis: a systematic review. Int J Tuberc Lung Dis. 2007;11(5):485–95. . 17439669

[61] World Health Organization. Same-day diagnosis of tuberculosis by microscopy. Geneva: WHO, 2011 Contract No.: WHO/HTM/TB/2011.7 23586121

[62] Ministry of Health and Family Welfare. Report of the First National Anti-Tuberculosis Drug Resistance Survey (2014–16). New Delhi, India: Ministry of Health and Family Welfare, 2018.

[63] Chiang SS, Swanson DS, Starke JR. New Diagnostics for Childhood Tuberculosis. Infect Dis Clin North Am. 2015;29(3):477–502. 10.1016/j.idc.2015.05.011 . 26188605

[64] Detjen AK, DiNardo AR, Leyden J, Steingart KR, Menzies D, Schiller I, et al Xpert MTB/RIF assay for the diagnosis of pulmonary tuberculosis in children: a systematic review and meta-analysis. Lancet Respir Med. 2015;3(6):451–61. 10.1016/S2213-2600(15)00095-8 . 25812968 PMC4756280

[65] MacPherson P, Houben R, Glynn JR, Corbett EL, Kranzer K. Pre-treatment loss to follow-up in tuberculosis patients in low- and lower-middle-income countries and high-burden countries: a systematic review and meta-analysis. Bull World Health Organ. 2014;92(2):126–38. 10.2471/BLT.13.124800 . 24623906 PMC3949536

[66] Cox H, Dickson-Hall L, Ndjeka N, Van't Hoog A, Grant A, Cobelens F, et al Delays and loss to follow-up before treatment of drug-resistant tuberculosis following implementation of Xpert MTB/RIF in South Africa: A retrospective cohort study. PLoS Med. 2017;14(2):e1002238 10.1371/journal.pmed.1002238 . 28222095 PMC5319645

[67] Shewade HD, Kokane AM, Singh AR, Verma M, Parmar M, Chauhan A, et al High pre-diagnosis attrition among patients with presumptive MDR-TB: an operational research from Bhopal district, India. BMC Health Serv Res. 2017;17(1):249 10.1186/s12913-017-2191-6 . 28376789 PMC5379759

[68] Shewade HD, Govindarajan S, Thekkur P, Palanivel C, Muthaiah M, Kumar AM, et al MDR-TB in Puducherry, India: reduction in attrition and turnaround time in the diagnosis and treatment pathway. Public Health Action. 2016;6(4):242–6. 10.5588/pha.16.0075 . 28123961 PMC5176048

[69] Nair D, Navneethapandian PD, Tripathy JP, Harries AD, Klinton JS, Watson B, et al Impact of rapid molecular diagnostic tests on time to treatment initiation and outcomes in patients with multidrug-resistant tuberculosis, Tamil Nadu, India. Trans R Soc Trop Med Hyg. 2016;110(9):534–41. 10.1093/trstmh/trw060 . 27738284

[70] Singla N, Satyanarayana S, Sachdeva KS, Van den Bergh R, Reid T, Tayler-Smith K, et al Impact of introducing the line probe assay on time to treatment initiation of MDR-TB in Delhi, India. PLoS ONE. 2014;9(7):e102989 10.1371/journal.pone.0102989 . 25058124 PMC4109962

[71] Botha E, den Boon S, Lawrence KA, Reuter H, Verver S, Lombard CJ, et al From suspect to patient: tuberculosis diagnosis and treatment initiation in health facilities in South Africa. Int J Tuberc Lung Dis. 2008;12(8):936–41. . 18647454

[72] Dunbar R, Lawrence K, Verver S, Enarson DA, Lombard C, Hargrove J, et al Accuracy and completeness of recording of confirmed tuberculosis in two South African communities. Int J Tuberc Lung Dis. 2011;15(3):337–43. . 21333100

[73] Mehra D, Kaushik RM, Kaushik R, Rawat J, Kakkar R. Initial default among sputum-positive pulmonary TB patients at a referral hospital in Uttarakhand, India. Trans R Soc Trop Med Hyg. 2013;107(9):558–65. 10.1093/trstmh/trt065 . 23920324

[74] Gopi PG, Chandrasekaran V, Subramani R, Narayanan PR. Failure to initiate treatment for tuberculosis patients diagnosed in a community survey and at health facilities under a DOTS program in a district of south India. Indian J Tuberc. 2005;52:153–6.

[75] Sreeramareddy C, Panduru K, Menten J, Van den Ende J. Time delays in diagnosis of pulmonary tuberculosis: a systematic review of literature. BMC Infect Dis. 2009;9(1):91. 19519917 10.1186/1471-2334-9-91 PMC2702369

[76] World Health Organization. Definitions and reporting framework for tuberculosis—2013 revision (updated December 2014). Geneva: WHO, 2014 Contract No.: WHO/HTM/TB/2013.2.

[77] Liu X, Lewis JJ, Zhang H, Lu W, Zhang S, Zheng G, et al Effectiveness of Electronic Reminders to Improve Medication Adherence in Tuberculosis Patients: A Cluster-Randomised Trial. PLoS Med. 2015;12(9):e1001876 10.1371/journal.pmed.1001876 . 26372470 PMC4570796

[78] WHO Country Office for India. Standards for TB Care in India. New Delhi: World Health Organization, 2014.

[79] Central TB Division. Revised National TB Control Programme Technical and Operational Guidelines for Tuberculosis Control in India. New Delhi: Government of India Ministry of Health and Familiy Welfare, 2016.

[80] Thomas A, Gopi PG, Santha T, Chandrasekaran V, Subramani R, Selvakumar N, et al Predictors of relapse among pulmonary tuberculosis patients treated in a DOTS programme in South India. Int J Tuberc Lung Dis. 2005;9(5):556–61. . 15875929

[81] Cox HS, Morrow M, Deutschmann PW. Long term efficacy of DOTS regimens for tuberculosis: systematic review. BMJ. 2008;336(7642):484–7. 10.1136/bmj.39463.640787.BE . 18250104 PMC2258398

[82] Kolappan C, Subramani R, Karunakaran K, Narayanan PR. Mortality of tuberculosis patients in Chennai, India. Bull World Health Organ. 2006;84(7):555–60. . 16878229 10.2471/blt.05.022087 PMC2627396

[83] Kolappan C, Subramani R, Kumaraswami V, Santha T, Narayanan PR. Excess mortality and risk factors for mortality among a cohort of TB patients from rural south India. Int J Tuberc Lung Dis. 2008;12(1):81–6. . 18173882

[84] Imperial MZ, Nahid P, Phillips PPJ, Davies GR, Fielding K, Hanna D, et al A patient-level pooled analysis of treatment-shortening regimens for drug-susceptible pulmonary tuberculosis. Nat Med. 2018;24(11):1708–15. 10.1038/s41591-018-0224-2 . 30397355 PMC6685538

[85] Narayanan S, Swaminathan S, Supply P, Shanmugam S, Narendran G, Hari L, et al Impact of HIV infection on the recurrence of tuberculosis in South India. J Infect Dis. 2010;201(5):691–703. 10.1086/650528 . 20121433

[86] Charalambous S, Grant AD, Moloi V, Warren R, Day JH, van Helden P, et al Contribution of reinfection to recurrent tuberculosis in South African gold miners. Int J Tuberc Lung Dis. 2008;12(8):942–8. . 18647455

[87] Verver S, Warren RM, Beyers N, Richardson M, van der Spuy GD, Borgdorff MW, et al Rate of reinfection tuberculosis after successful treatment is higher than rate of new tuberculosis. Am J Respir Crit Care Med. 2005;171(12):1430–5. 10.1164/rccm.200409-1200OC . 15831840

[88] Nunn AJ, Phillips PP, Mitchison DA. Timing of relapse in short-course chemotherapy trials for tuberculosis. Int J Tuberc Lung Dis. 2010;14(2):241–2. . 20074418

[89] Stop TB Partnership. REACH launches video series with TB survivors and Champions 2017. Available from: http://www.stoptb.org/news/frompartners/2017/fp17_040.asp. [cited 2018 Nov 6]

[90] Jackson C, Stagg HR, Doshi A, Pan D, Sinha A, Batra R, et al Tuberculosis treatment outcomes among disadvantaged patients in India. Public Health Action. 2017;7(2):134–40. 10.5588/pha.16.0107 . 28695087 PMC5493095

[91] Alipanah N, Jarlsberg L, Miller C, Linh NN, Falzon D, Jaramillo E, et al Adherence interventions and outcomes of tuberculosis treatment: A systematic review and meta-analysis of trials and observational studies. PLoS Med. 2018;15(7):e1002595 10.1371/journal.pmed.1002595 . 29969463 PMC6029765

[92] Uplekar M, Weil D, Lonnroth K, Jaramillo E, Lienhardt C, Dias HM, et al WHO's new end TB strategy. Lancet. 2015;385(9979):1799–801. 10.1016/S0140-6736(15)60570-0 . 25814376

