# Supplementary material for: Constructing care cascades for active tuberculosis: A strategy for program monitoring and identifying gaps in quality of care
Source: PLoS Med. 2019 Feb 27;16(2):e1002754. doi: 10.1371/journal.pmed.1002754 (PMC6392267; doi:10.1371/journal.pmed.1002754)
Supplement: S1 Appendix — (PDF) [file pmed.1002754.s001.pdf]

Supplementary Appendix: Constructing a tuberculosis cascade of care: a “how to” guide

Supplement to:

Constructing care cascades for active tuberculosis: a strategy for program monitoring and identifying gaps in quality of care

Corresponding author:

Ramnath Subbaraman, MD, MSc, FACP
Assistant Professor
Department of Public Health and Community Medicine
Tufts University School of Medicine
Division of Geographic Medicine and Infectious Diseases
Tufts Medical Center
136 Harrison Avenue, MV237
Boston, MA 02111
Email: ramnath.sub@gmail.com

Constructing a tuberculosis cascade of care: a "how to" guide

This document is a practical guide to assist tuberculosis (TB) program managers and researchers in constructing TB care cascades that are relevant and appropriate to their local settings. As a result, this document provides relatively simplified approaches for achieving cascade estimates. We describe two different approaches below. The methodological strengths and limitations of each approach are discussed in the section of the main manuscript titled "General principles for constructing a cascade." We encourage modification of these approaches as needed for each setting.

The first approach, which we will refer to as the *routine data approach*, is a denominator-numerator unlinked methodology [1], in which the estimates for each stage are gathered or extrapolated from readily available programmatic data or previously conducted local studies. The routine data approach depends on the availability of robust data collection by TB programs and of previously conducted studies to estimate specific gaps in the cascade. If such information is available, the primary data collection and data analysis requirements for the routine data approach are substantially less demanding than for the cohort-based approach. This general approach has been used most recently to estimate TB care cascades at a national level for India and South Africa [2,3]. However, this approach may introduce bias into cascade estimates, because it fails to account for the changing composition of the patient population at each stage of the cascade. Additionally since this approach involves the retrospective use of data, it does not enable evaluation of reasons for gaps in the care cascade.

The second approach, which we will refer to as the *cohort-based approach*, uses a denominator-denominator linked method, in which the same group of individuals is followed across multiple stages of the cascade. In practice, this approach requires prospective or retrospective cohort studies that can be conducted in TB programs with high-quality patient records. The cohort-based approach is more resource intensive, because it requires primary data collection; however, this approach may minimize bias in cascade estimates.

We recommend that estimates be calculated independently for different forms of TB, depending on the primary diagnostic modality used in a given setting. For example, sputum smear microscopy is still the initial diagnostic test used in India, which is a relatively low HIV prevalence country. A recent evaluation of the TB care cascade in India provided separate estimates for new smear-positive, new smear-negative, extrapulmonary, retreatment smear-positive, retreatment smear-negative, and multidrug-resistant (MDR) TB patients—following definitions of these patient categories used by India's Revised National TB Control Programme [2].

In contrast, South Africa is a country with high HIV prevalence, and Xpert MTB/Rif is a commonly used diagnostic test, in addition to smear microscopy and (to a lesser extent) mycobacterial culture. A recent evaluation of the TB care cascade in South Africa provided separate estimates for drug-susceptible TB patients, HIV-infected drug-susceptible TB patients, and rifampin-resistant TB patients (i.e., patients presumed to have MDR TB) [3]. These groups included combined estimates for patients with a positive bacteriological test (i.e, Xpert- or smear-positive) and patients with negative bacteriological tests (i.e., Xpert- or smear-negative).

Approach 1: the routine data approach

Step A: Number of individuals registered in TB treatment

The first step is to obtain data on the number of individuals who are *registered in TB treatment* (cascade Step 4) for different types of TB, over a one-year time period (Figure 1).

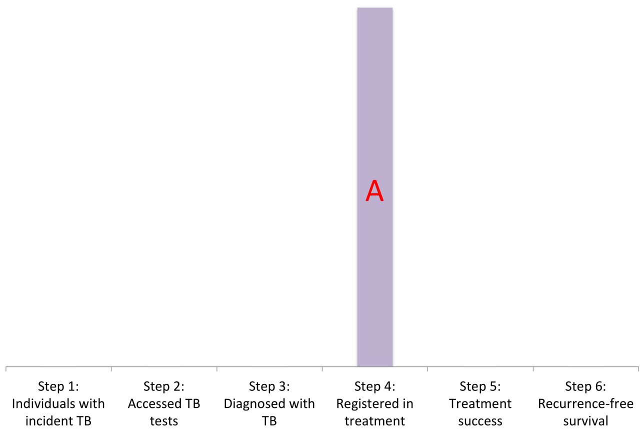

Figure 1: Step A in the routine data approach to constructing a TB care cascade

These statistics can be obtained from a variety of sources, including hospital or clinic records or city- or district-level TB reports to construct local-level cascades. For country-level cascades, these statistics can be obtained from national reports (e.g., *TB India* reports for India) [4,5] or from national electronic databases with individual-level data (e.g., South Africa's electronic TB and drug-resistant TB registers) [3]. In addition, the World Health Organization (WHO) reports this information for several countries in its case notifications database (http://www.who.int/tb/country/data/download/en/) (Table 1).

Table A. Variables in the WHO TB case notifications database that provide estimates of the number of individuals registered in TB treatment at a country level

Variable	Types of patients included
new_labconf	New pulmonary bacteriologically confirmed TB cases (e.g., smear-, Xpert-, or culture-positive)
new_clindx	New pulmonary clinically diagnosed TB cases (not bacteriologically confirmed)
new_ep	New extrapulmonary cases (bacteriologically confirmed or clinically diagnosed)
ret_rel_labconf	Relapse pulmonary bacteriologically confirmed TB cases (e.g., smear-, Xpert-, or culture-positive)
ret_rel_clindx	Relapse pulmonary clinically diagnosed TB cases (not bacteriologically confirmed)
ret_rel_ep	Relapse extrapulmonary cases (bacteriologically confirmed or clinically diagnosed)
ret_nrel	Previously treated patients, excluding relapse cases (pulmonary or extrapulmonary, bacteriologically confirmed or clinically diagnosed)
conf_rrmdr_tx	Rifampin-resistant (RR) or MDR TB patients who were laboratory confirmed and started on treatment
conf_xdr_tx	Extensively drug-resistant (XDR) TB patients who were laboratory confirmed and started on treatment

To start with, we suggest separately extracting treatment registration numbers for each of the following forms of TB, at minimum: (1) laboratory confirmed new pulmonary TB patients (i.e., smear-, Xpert-, or culture-positive cases); (2) empirically diagnosed new pulmonary TB patients (i.e., smear-, Xpert-, or culture-negative cases); (3) new extrapulmonary TB patients; (4) laboratory confirmed retreatment pulmonary TB patients; (5) empirically diagnosed retreatment pulmonary TB patients and retreatment extrapulmonary TB patients (sometimes referred to collectively as “retreatment other” patients); and (6) drug-resistant TB patients (including MDR and extensively drug-resistant, or XDR, TB patients). Individual cascades can then be constructed for each of these different types of TB. These individual cascades can later be easily combined to construct a cascade that includes all forms of TB.

Step B: Number of individuals who achieve treatment success

The second step is to obtain data on the number of patients who achieve *treatment success* (cascade Step 5) for different types of TB, over the same one-year time period (Figure 2).

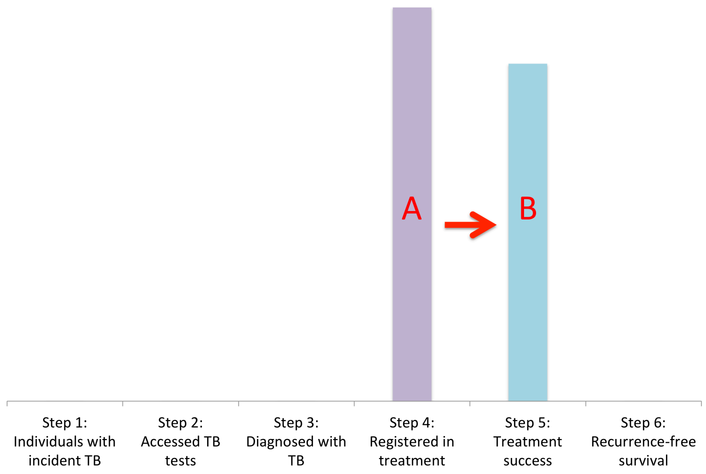

Figure 2: Step B in the routine data approach to constructing a TB care cascade

These statistics can be obtained from similar sources as listed for Step A, including clinic records or city- and district-level TB reports to construct local-level cascades. For country-level cascades, these statistics can be obtained from national reports (e.g., the *TB India* reports for India) [4,5] or from national electronic TB databases with individual-level data (e.g., South Africa’s electronic TB and drug-resistant TB registers) [3]. The World Health Organization (WHO) aggregates and reports this information for several countries in its “treatment outcomes” database (http://www.who.int/tb/country/data/download/en/), though the categorization of types of TB and the variables under which treatment success data are captured in the WHO database have varied over time.

We recommend extracting treatment success data that correspond to the categories described above: (1) laboratory confirmed new pulmonary TB patients; (2) empirically diagnosed new pulmonary TB patients; (3) new extrapulmonary TB patients; (4) laboratory confirmed retreatment pulmonary TB patients; (5) empirically diagnosed retreatment pulmonary and retreatment extrapulmonary TB patients

(sometimes referred to collectively as "retreatment other" patients); and (6) drug-resistant TB patients (including MDR and XDR patients).

Step C: Number of individuals who achieve one-year recurrence-free survival

The third step is to estimate the number of individuals who *achieve TB recurrence-free survival for at least one-year* (cascade Step 6) after the completion of TB therapy (Figure 3). Most TB programs do not routinely engage in long-term follow-up of patients after the completion of TB treatment; however, studies that estimate post-treatment TB disease recurrence and mortality rates in your specific country or region may be available in the published literature (Table B).

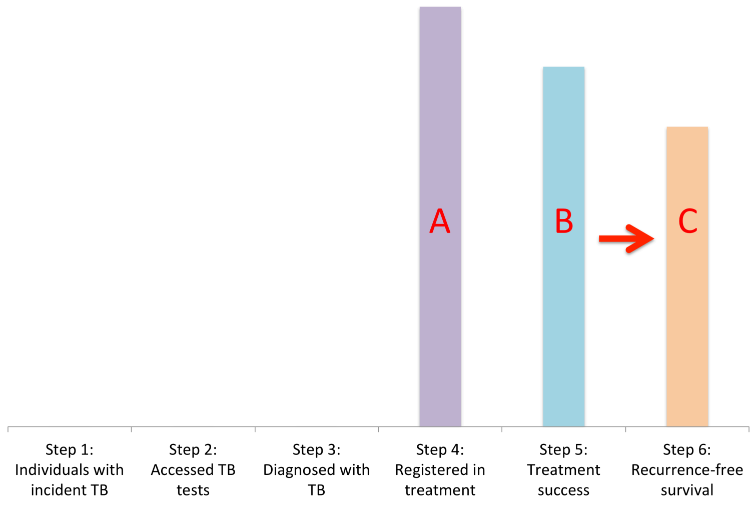

Figure 3: Step C in the routine data approach to constructing a TB care cascade

Table B: Examples of studies estimating post-treatment tuberculosis recurrence rates under routine programmatic conditions

Author	Country	Year of cohort	Post-treatment recurrence and death rates
Becerra et al. [6]	Peru	1999—2002	5% for MDR TB patients
Cox et al. [7]	Uzbekistan	2001—2002	7% for drug-susceptible TB patients; 44% for MDR TB patients
Gelmanova et al. [8]	Russia	2000—2004	7% for MDR TB patients
Marx et al. [9]	South Africa	1996—2008	16.5% of smear-positive TB patients
Sadacharam et al. [10]	India	2002—2003	18% of new smear-positive TB patients; 27% of previously treated TB patients
Thomas et al. [11]	India	2000—2001	15% of new smear-positive TB patients
Velayutham et al. [12]	India	2015—2016	11% of new smear-positive TB patients experienced TB recurrence within 12 months; ~2% died

It is important that estimates of post-treatment recurrence and death be extracted from observational studies conducted under programmatic conditions (i.e., routine care provide by TB programs), rather than from rigorous clinical trials, since clinical trials may reflect a higher standard of care than is normally delivered under programmatic conditions. For example, a systematic review of studies of post-treatment TB recurrence for patients taking the DOTS regimen found considerably higher TB recurrence rates for patients treated under routine programmatic conditions [7].

Using your local estimate of post-treatment TB recurrence and death, you can then estimate Step 6 of the cascade as follows:

Step 6 cascade value = (Step 5 cascade value) – (estimated post-treatment TB recurrence and death rate)(Step 5 cascade value)*

In settings where estimates of post-treatment TB recurrence and death are not available, there are two options. First, you can conduct a study using representative sampling of patients or health centers to estimate the local rate of post-treatment TB recurrence and death, which can be retrospective [6,8-10,13] or prospective [11,12]. If a prospective approach is used, we recommend following the rigorous methodology used by Velayutham et al. [12]. In that study, a cohort of Indian patients who completed TB therapy under programmatic conditions were followed prospectively with follow-up visits by researchers every 3 months. During these visits, patients were screened for symptoms of TB and sputum samples were collected for sputum microscopy and mycobacterial culture to help diagnose recurrent TB. This methodology minimized post-treatment loss to follow-up of patients, screened systematically for TB recurrence, and also carefully captured information on mortality. Post-treatment mortality should be included as a suboptimal outcome in Gap 5, because studies suggest that the increased risk for mortality in TB patients extends for several months after the completion of TB treatment, potentially due to disease relapse, undiagnosed drug-resistant, or pulmonary complications (e.g., fibrosis and bronchiectasis) of TB [14,15]. As such, death in the year after completing TB treatment may also reflect the quality of care delivered during TB therapy.

If it is not possible to conduct a local study of TB recurrence, then Step 6 of the TB cascade can be omitted, which results in treatment success (Step 5) being the final step of the cascade.

Step D: Number of individuals diagnosed with TB

Next, we move “backwards” from the number of individuals who are registered in TB treatment (cascade Step 4) to estimate the preceding steps of the cascade, starting with estimation of the number of individuals *diagnosed with TB* (cascade Step 3) (Figure 4).

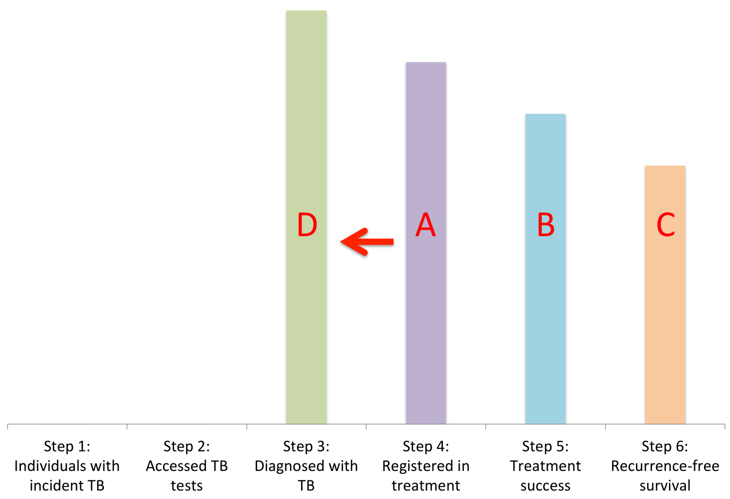

Figure 4: Step D in the routine data approach to constructing a TB care cascade

The number of individuals diagnosed with TB can be estimated using a few approaches. In some countries, diagnosed TB patients are immediately "notified" to TB programs. These TB programs typically have electronic records of all diagnosed TB patients. South Africa uses an electronic system with unique patient identifier numbers enabling patients to be followed through multiple stages of the cascade [13]. Other TB programs report the number of diagnosed patients in aggregate for some forms of TB; however, they do not capture individual records of diagnosed patients. For example, India reports the aggregate "number of smear-positive patients diagnosed" every year at the district and national levels (notably, these values are only reported for smear-positive patients and not for other forms of TB) [4]. Unlike electronic databases containing individual-level data, this information does not allow tracking of individual patients through each subsequent step of the cascade.

In situations where individual-level or aggregate data on the number of diagnosed TB patients are not available, we recommend estimating the number of diagnosed TB patients (cascade Step 3) by back-calculation from the number of patient registered for TB therapy (cascade Step 4) using estimates of pretreatment loss to follow-up (i.e., the number of diagnosed TB patients who fail to get registered for TB therapy).

In addition to targeted searches of the medical literature, a few resources are available to help identify studies of pretreatment loss to follow-up that may be relevant for your country, region, district, or city. First, a systematic review published in 2014 summarized findings from 23 studies of pretreatment loss to follow-up conducted throughout countries in Africa, Asia, and the Western Pacific region (Table C) [16]. Second, the recently published Indian and South African TB care cascades both estimated pretreatment loss to follow-up through systematic reviews of local studies in those two countries [2,3]. Individual local studies contained in these systematic reviews may be helpful for estimating subnational cascades in these two countries. In some situations, studies that estimate pretreatment loss to follow-up at the national level may be available, as is the case for MDR TB patients in South Africa [13].

Table C: Examples of studies estimating TB pretreatment loss to follow-up rates in different settings

Author	Country	Scope of study	Year of data collection	Pretreatment loss to follow-up rate
Subbaraman et al. [2]	India	Systematic review of 16 local Indian studies	2000—2015	16% for new patients; 23% for MDR TB patients
Naidoo et al. [3]	South Africa	Systematic review of 15 local South African studies, including drug-susceptible TB	2006—2016	19% for bacteriologically confirmed patients
Cox et al. [13]	South Africa	Nationally-representative study of rifampin-resistant (presumed MDR TB) patients in South Africa	2011, 2013	37% for MDR TB patients after widespread introduction of Xpert testing
Uchenna et al. [17]	Nigeria	Five states in southern Nigeria	2009	17% for smear-positive patients
Razia et al. [18]	Pakistan	Five tertiary centers and 16 peripheral centers in one district	2009	6% for smear-positive patients
Buu et al. [19]	Vietnam	Several district tuberculosis units	2000	8% for smear-positive patients
Korobitsyn et al. [20]	Tajikistan	Four districts	2008-2009	8% for smear-positive patients

In some cases, studies of pretreatment loss to follow-up that are relevant to your country, region, district, or city may not be available. In those situations, new studies can be designed and conducted to estimate pretreatment loss to follow at the clinic, city, district, regional, or national level. For countries interested in estimating pretreatment loss to follow-up at the national level, we recommend using rigorous representative sampling approaches (e.g., probability proportionate to size) to select clinics or districts throughout the country where these studies can be conducted. In addition, rigorous prospective studies with careful patient tracking strategies are most likely to achieve accurate estimates of pretreatment loss to follow-up.

Once estimates of pretreatment loss to follow-up are determined for each form of TB, you can then estimate Step 3 of the cascade as follows:

Step 3 cascade value = (Step 4 cascade value) / (1 – estimated pretreatment loss to follow-up rate)

Step E: Number of individuals with TB who reached health facilities and accessed a TB diagnostic test

Next, we move "backwards" from the number of patients who are *diagnosed with TB* (cascade Step 3) to estimate the *number of TB patients who accessed a TB diagnostic test (or who had an appropriate diagnostic workup initiated for extrapulmonary TB)* (cascade Step 2) (Figure 5).

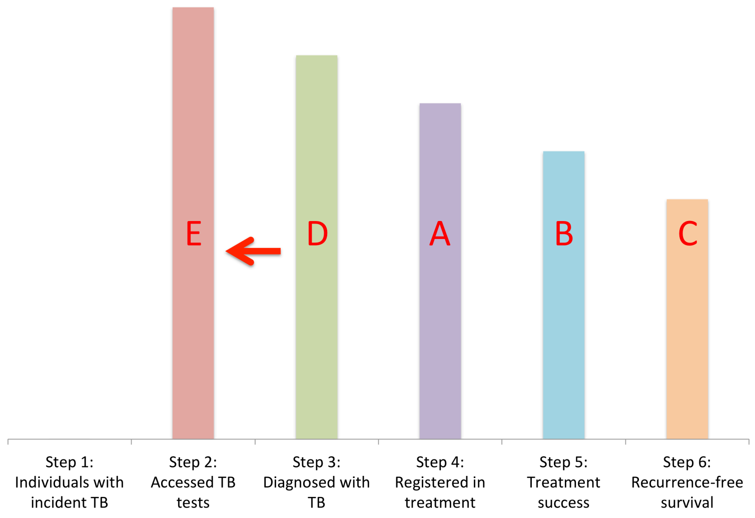

Figure 5: Step E in the routine data approach to constructing a TB care cascade

Estimation of this step requires different approaches for every form of TB, and we describe approaches to estimating Step 2 separately for each form of TB.

Estimating Step 2 for pulmonary TB patients who would have a positive bacteriological test

For pulmonary TB patients who would have a positive bacteriological test (i.e., smear- or Xpert-positive) if they completed an evaluation for TB, an estimation of the proportion of patients who are drop out between Step 2 and Step 3, that incorporates a measure of the accuracy of the diagnostic test used, can facilitate back-calculation of the value for Step 2 from the value for Step 3.

For example, India primarily uses sputum microscopy for upfront diagnosis of most pulmonary TB patients. Two sputum samples are collected and tested on separate days—a "spot" sample at the time of initial evaluation and a second "morning" sample the next day. In the Indian context, one of the ways in which a smear-positive patient could reach a TB diagnostic facility and access a TB test (Step 2) but remain undiagnosed would be if she had a false negative "spot" sample and did not return to the clinic the next day to submit a second "morning" sample that would have resulted in a diagnosis. In the recently published Indian cascade of care, the authors estimated that approximately 11% of patients visiting TB microscopy centers do not submit a second sputum sample for evaluation, based on a systematic review and meta-analysis of studies [2]. A recent meta-analysis found that the incremental yield of a second sputum sample for diagnosing smear-positive TB is 11.9% [21]. Using these two values, the authors first estimated the proportion of smear-positive patients who go undiagnosed as follows:

Proportion of smear-positive patients who go undiagnosed = (Proportion of all patients screened who submit one sputum sample but do not submit a second sputum sample)(Incremental yield of a second sputum sample for diagnosing smear-positive TB)*

Using this value for the proportion of smear-positive patients who go undiagnosed, it is then possible to estimate the value for cascade Step 2 for smear-positive patients as follows:

Step 2 cascade value for smear-positive patients = (Step 3 cascade value for smear-positive patients) / (1 – proportion of smear positive patients who go undiagnosed)

To estimate the proportion of Xpert-positive TB patients who go undiagnosed, a similar approach may be used to estimate Step 2 in settings that primarily use Xpert for upfront testing, since a small percentage of patients may initially have Xpert test results that return as "error," "invalid," or "no result." Some of these patients may have a positive test result if Xpert testing is repeated, which may be standard practice in some settings. However, it is worth noting that, in general, a fairly small percentage of all sputum samples (~1%) [22] return with an error or invalid result, so it may be reasonable to skip these calculations and to simply assume that there are no Xpert-positive TB patients who go undiagnosed. Under this assumption, the Step 2 estimate is equal to the Step 3 estimate for Xpert-positive patients.

<u>Estimating Step 2 for pulmonary TB patients who have a negative bacteriological test (i.e. diagnosed empirically)</u>

Estimating the number of patients who have a negative bacteriological test (i.e., negative sputum microscopy or negative Xpert result) but who would truly have pulmonary TB (if tested with a more sensitive test such as mycobacterial culture or if diagnosed empirically after a rigorous workup) is very challenging. Admittedly any estimates produced may have considerable uncertainty. However, estimation of Step 2 for patients who have a negative bacteriological test can provide some of the most useful information in the entire TB cascade, since these estimates help to evaluate the efficiency of protocols for empiric diagnosis of patients in a TB program.

For example, in the Indian TB cascade, the authors estimated that about 514,161 patients who truly had smear-negative TB likely reached government TB facilities and accessed a TB test in India; however, only 320,982 smear-negative patients were estimated to have been successfully diagnosed at government TB facilities. This suggested that 193,179 smear-negative patients, or about 38% of all smear-negative TB patients reaching government TB diagnostic facilities and accessing TB tests, were not being successfully diagnosed—highlighting considerable inefficiency in processes for empiric diagnosis of TB patients [2].

One approach is to estimate the number of true smear- or Xpert-negative patients who reached TB diagnostic facilities and accessed a TB test by extrapolation from the number of smear- or Xpert-positive patients who accessed a TB test—since the number of patients with positive tests is generally a more reliable estimate. This extrapolation can be calculated using estimates of the sensitivity of sputum smear microscopy or Xpert (Table D).

Table D: Estimates of the sensitivity of diagnostic tests for pulmonary TB

Author	Scope of study	Year of data collection	Estimated sensitivity for culture-positive TB
Sputum smear microscopy			
Davis et al. [23]	Systematic review of 8 studies comparing sputum microscopy with samples collected over multiple days with same-day microscopy	Studies published from 2005—2012	64% for multi-day sputum microscopy vs. 63% for same-day microscopy
Steingart et al. [24]	Systematic review of 45 studies comparing conventional Ziehl-Neelsen or Kinyoun sputum microscopy with fluorescent microscopy	Studies published from 1950—2004	32% to 94% for conventional microscopy vs. 52% to 97% with fluorescent microscopy
Subbaraman et al. [2]	Smear sensitivity estimate extrapolated from study of Xpert MTB/Rif implementation across 18 geographically diverse sites in India[25]	2012—2013	59% (95%CI:56% to 61%) for smear microscopy as used throughout the public sector in India
Xpert MTB/Rif			
Steingart et al. [22]	Systematic review and meta-analysis of 27 studies	Studies published up to 2013	86% in HIV-negative patients; 79% in HIV-positive patients
Xpert Ultra			
Dorman et al. [26]	10 sites across 8 high burden countries	2016	91% in HIV-negative patients; 90% in HIV-positive patients

Since the sensitivity of these tests may vary by country, region, or health facility—especially for sputum smear microscopy [24]—ideally, relevant local studies should be used. For example, the Indian TB cascade estimated the sensitivity of sputum smear microscopy based on a recent nationally representative study of the rollout of Xpert, which allowed the authors to estimate that sputum smear microscopy has approximately 59% sensitivity in India [2]. In cases where robust local studies are not available, estimates of the sensitivity of these tests from multi-national studies [26] or from systematic reviews and meta-analyses can be used (Table D) [22-24,27].

Using the example of sputum microscopy, once the correct sensitivity estimate is determined, the number of smear-negative TB patients in Step 2 can be estimated as follows:

Step 2 cascade value for smear-negative patients = (Step 2 cascade value for smear-positive patients)(1 – sensitivity) / (sensitivity)*

Similarly, the number of Xpert-negative TB patients in Step 2 can be estimated as follows:

Step 2 cascade value for Xpert-negative patients = (Step 2 cascade value for Xpert-positive patients)(1 – sensitivity) / (sensitivity)*

An alternative (or potentially complementary) approach to inform estimates of Step 2 and Gap 2 for smear-negative or Xpert-negative patients could be to quantify the number of patients evaluated at TB

diagnostic facilities who ultimately receive a medical diagnosis. While there may be uncertainty about the true number of smear- or Xpert-negative patients undergoing evaluation, ideally, if the medical care is of high quality, all patients who are evaluated for suspected TB should receive a TB- or non-TB-related medical diagnosis (e.g., community-acquired pneumonia, upper respiratory infection, chronic obstructive pulmonary disease) and appropriate follow-up plan. If most patients with negative sputum smears or Xpert results do not get evaluated further and do not receive a medical diagnosis, this may suggest poor quality of medical care more generally. There are substantial limitations to this approach, however, given the considerable challenges in diagnosing smear- or Xpert-negative TB in settings without access to mycobacterial culture. In addition, encouraging designation of a medical diagnosis for all patients may provide healthcare workers with an incentive to label patients as having common non-TB pulmonary conditions without completing the careful diagnostic workup required to rule out smear- or Xpert-negative TB.

Estimating Step 2 for extrapulmonary TB patients

Estimating Step 2 for extrapulmonary TB patients is very challenging. Ideally, this value would be informed by robust studies that estimate the proportion of extrapulmonary TB patients who get evaluated at TB diagnostic facilities but who fail to get appropriately diagnosed. Such estimates are very challenging to obtain in most low- and middle-income countries, where extrapulmonary TB is frequently diagnosed clinically without collection of diagnostic samples for mycobacterial stain, culture, polymerase chain reaction-based testing, or histopathology.

In addition, the ease of diagnosing extrapulmonary TB based on clinical grounds varies substantially based on the sites of disease. For example, TB lymphadenitis, especially involving neck lymph nodes, is generally visible and easy to diagnose. TB meningitis is usually serious enough to warrant hospitalization, which may facilitate its diagnosis. In contrast, TB pleuritis, miliary TB, and TB at other body sites generally require a chest X-ray or more advanced imaging to facilitate diagnosis, and these imaging modalities may not be widely accessible in many low- and middle-income countries.

In light of these challenges, we suggest two potential approaches to estimating Step 2 for extrapulmonary TB patients. The first approach would be to conduct robust studies at TB facilities in which patients with suspected extrapulmonary TB are followed prospectively through the entire workup, with the goal of estimating the proportion who do not complete the clinical workup to achieve a diagnosis. These studies could be conducted at a single clinic or hospital (to construct a local cascade) or at a representative sample of health facilities (to construct regional or national cascades).

A second approach, used in the Indian TB care cascade [2], is to assume that extrapulmonary TB is more challenging to diagnose than smear-positive pulmonary TB but easier to diagnose than smear-negative TB, since some common forms of extrapulmonary TB are more clinically evident. Under these assumptions, the proportion of extrapulmonary TB patients who remain undiagnosed despite reaching a TB diagnostic facility and having an appropriate workup initiated by a health provider can be estimated by taking the average of the proportion of undiagnosed smear-positive TB patients and the proportion of undiagnosed smear-negative TB patients. This approach will likely yield a conservative estimate for the proportion of extrapulmonary TB patients who remain undiagnosed in most low- and middle-income countries.

Whenever possible, we recommend using the first approach (conducting prospective cohort studies) to estimate the proportion of extrapulmonary TB patients who may remain undiagnosed, because these prospective cohort studies provide real-world data to help estimate this gap.

Once an estimate is achieve for the proportion of extrapulmonary TB patients who had an appropriate workup initiated by a healthcare provider but who remain undiagnosed, then the Step 2 value for extrapulmonary TB patients can be estimated as follows:

Step 2 cascade value for extrapulmonary TB patients = (Step 3 cascade value for extrapulmonary TB patients) / (1 – proportion of extrapulmonary TB patients who go undiagnosed)

<u>Estimating Step 2 for MDR TB patients</u>

Step 2 for MDR (or rifampin-resistant, RR) TB patients can be arrived at using estimates of the number of MDR TB patients among notified pulmonary TB patients. These data are routinely reported by the WHO based either upon surveillance data from the National TB Programs in these countries or upon modeling estimates. The surveillance data estimates are generally derived from studies that screen all patients with suspected TB using mycobacterial culture at TB diagnostic facilities linked to national TB programs [28]. These surveillance data provide estimates of the proportion of MDR TB among new and retreatment pulmonary TB patients. Extrapolating from these proportions, the WHO is then able to estimate the probable number of MDR TB patients among all pulmonary TB patients who get diagnosed in the national TB programs in these different countries. These values are reported in online WHO datasets under variable names such as *e_rr_in_notified_pulm* (estimated number of rifampin-resistant TB cases among notified pulmonary TB cases).

Note that the estimated number of MDR TB patients among all pulmonary TB patients diagnosed is not the same as the number of MDR TB patients who are actually diagnosed by national TB programs. Because many national TB programs still primarily use sputum microscopy and many clinicians in the private sector diagnose TB empirically, many patients are not diagnosed (or even screened for) MDR TB and are instead misclassified as drug-susceptible TB patients, resulting in the need to use surveillance data to estimate the number of MDR-TB patients who reach TB diagnostic facilities and access a TB test (Step 2).

<u>Estimating Step 2 for children with TB</u>

A recent analysis of the care cascade for children in Uganda and Kenya estimated the number of children with active TB in the population using the TB case detection rate for children in Africa [29]. This approach does not account for variability in the quality of clinical workup and differences in case detection across African countries, and it does not allow for estimation of the proportion of patients who might be missed because of suboptimal adherence to clinical algorithms for empirical diagnosis. We therefore recommend estimating Stage 2 for children using similar methods as those described for adult TB patients, but with substitution of high-quality estimates of the sensitivity of different diagnostic tests in children [30,31], especially high-quality local estimates where available. This would allow for estimation of the number of smear-, Xpert-, or culture-negative children with TB who reach TB diagnostic facilities and accessed a test based on the number of bacteriologically-confirmed child TB patients. Comparing these estimates to the number of patients who are actually diagnosed empirically

would provide information on the number of children with sputum- or Xpert-negative active TB who may be missed during the diagnostic workup (Gap 2).

Step F: Number of individuals with incident or prevalent TB in the population

Step 1 in the TB cascade—estimating the overall number of individuals with incident or prevalent TB in the population—is arguably the most important step in cascade model (Figure 6). Having an estimate for Step 1 allows estimation of the number of TB patients who do not reach health facilities and access a TB test (Gap 1), which may be the largest gap in the TB cascade in some low- and middle-income countries [2].

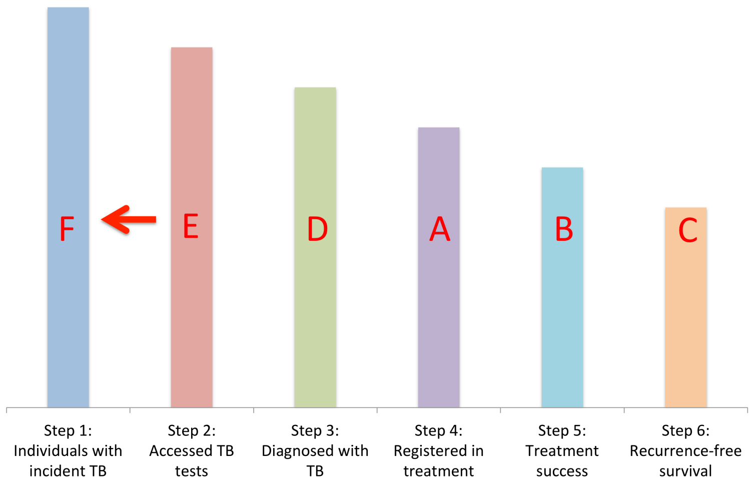

Figure 6: Step F in the routine data approach to constructing a TB care cascade

The ideal estimate of TB burden to use for constructing national- or regional-level cascade is the number of individuals with incident TB in a population. Incidence estimates are extremely challenging to arrive at, however, and these estimates often have considerable uncertainty. As such, we suggest two alternative approaches:

(1) Using estimates of TB burden from population-based point prevalence surveys of active TB as the value for Step 1. A point prevalence estimate for India was used in the initial estimation of the Indian TB cascade of care [2], and nationally-representative point prevalence survey data are available for some countries [32] and even some cities [33].

(2) In the South African TB cascade, the authors use the WHO time series analysis of TB incidence (which is usually estimated using data on changes in notification rates over time) to estimate the TB burden for all TB patients. The authors added the incident cases for a single year plus 50% of the undetected cases from the prior year, under the assumption that about half of undetected cases from the prior year would have died or achieved self-cure [3].

Other approaches for estimating TB incidence or collecting data on TB prevalence are mentioned in the main manuscript.

Approach 2: the cohort-based approach

Use of a cohort-based approach to constructing a TB care cascade has the potential to provide rigorous denominator-denominator linked estimates of patient losses across multiple cascade stages, though it may also be relatively resource-intensive, as it required primary data collection [1]. This approach can be used to evaluate local care cascades at a clinic, hospital, or city level.

This approach could also be used to achieve care cascade estimates at a regional or national level, if rigorous representative sampling (e.g., probability proportionate to size) is used to select clinics or districts throughout a country where prospective or retrospective data could be collected for patient cohorts at each site [34]. While Approach 2 (the cohort-based approach) is more time- and labor-intensive, it is likely to produce more accurate cascade estimates for national-level cascades than Approach 1 (the routine data approach). Serial cohort-based studies would allow use of the care cascade for assessment of changes in a national TB program's outcomes over time. Finally, cohort-based studies may be the only practical approach to estimating TB cascades in settings where pre-existing data on key gaps in care are limited.

We recommend that individuals with TB be tracked prospectively if possible, rather than tracking them retrospectively using health records. The benefits of tracking patients prospectively are as follows:

(1) Medical records (particularly paper records) often contain incomplete patient information. Researchers may be able to obtain more complete information when following patients prospectively, because healthcare providers are more likely to remember specific patient details.

(2) Tracking patients who have similar names through medical records can be very challenging if a study is conducted retrospectively.

(3) Determining the true outcomes for patients listed as having been "lost to follow-up" can be very challenging or impossible if patients are tracked retrospectively months or years after these outcomes have occurred. For example, pretreatment loss to follow-up patients (i.e., patients diagnosed with TB who do not start TB treatment) may have actually started on TB treatment at another facility. Patients on treatment who are reported as being lost to follow-up may have actually transferred care to another TB facility. If researchers are trying to determine post-treatment TB recurrence rates, contacting patients months or years after TB treatment has been completed may be impossible, since they may have moved to other locations or changed their contact information. If patients are followed prospectively, researchers are more likely to be able to track patients and contact them directly to determine their true outcomes.

Steps A, B, C, and D: tracking a single cohort of patients from TB diagnosis to post-treatment recurrence-free survival

Step A in the cohort-based approach is to identify all patients diagnosed with TB (cascade Step 3) at the selected TB diagnostic facilities (Figure 7). Ideally, this initial cohort should include patients diagnosed with all forms of TB—bacteriologically-diagnosed pulmonary TB, empirically-diagnosed pulmonary TB (i.e., those diagnosed with TB without a positive bacteriological test), extrapulmonary TB, and MDR TB. As noted above, these diagnosed patients should ideally be identified in a prospective fashion to facilitate tracking and determination of their outcomes throughout the subsequent stages of the cascade.

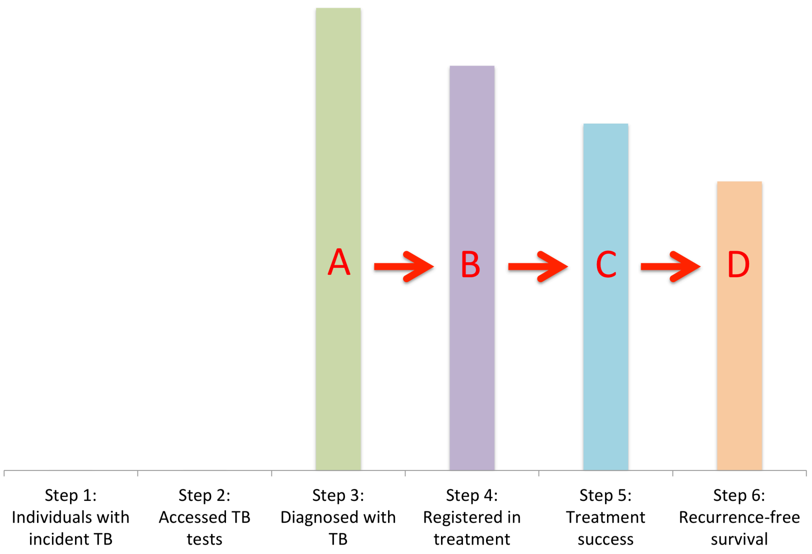

Figure 7: Steps A, B, C, and D in the cohort-based approach to constructing a TB care cascade

For Step B, these patients diagnosed with TB can then be tracked to determine who got successfully registered for and started on TB treatment (cascade Step 4). One challenge that arises in Step B is defining the length of time that can elapse between a patient's diagnosis and treatment registration before she is classified as a pretreatment loss to follow-up case. For example, in a meta-analysis of 14 studies of pretreatment loss to follow-up conducted in India, the different studies variably defined pretreatment loss to follow-up as consisting of patients who did not get registered for treatment between 2 weeks to as long as 3 months after the date of diagnosis [2]. A retrospective study in South Africa defined pretreatment loss to follow-up as consisting of patients who did not get registered for treatment within 6 months of diagnosis [13].

This decision regarding the "elapse time" required to define pretreatment loss to follow-up will depend on the study methodology used. For example, for studies in which patients are tracked prospectively to determine outcomes, research teams are often ethically obliged to intervene to retrieve patients who have not successfully registered to get them started on TB treatment, so a shorter elapse time (e.g., two to four weeks) may be reasonable. For retrospective studies that happen months to years after patients are diagnosed, longer elapse times (e.g., three to six months) may provide a more accurate estimate of how many patients do not get registered in TB treatment even with a relatively long duration of follow-up. In rare situations where rigorous longitudinal patient databases are available, it may be possible to estimate this step using rigorous survival methodologies, which can estimate time delays and

pretreatment loss to follow-up rates, as was done for a HIV care cascade study conducted in KwaZulu-Natal, South Africa [34].

For Step C, the patients in the original cohort who successfully get registered in TB treatment can then be followed to determine the proportion who achieve treatment success (cascade Step 5). This step may be relatively easy to estimate in most settings, because most national TB programs carefully document treatment outcomes for individual TB patients. In addition, with careful retrospective audits of individual treatment cards, survival methodologies can be also be used to present these findings, which may allow visualization of the relative time points during the treatment course when most patients experience unfavorable outcomes (i.e., loss to follow-up, treatment failure, or death). In settings where a large proportion of TB patients are treated in the private sector, TB drug sales data or the use of vouchers for patient medication refills at private pharmacies in public-private interface initiatives may facilitate estimation of this cascade stage [35].

For Step D, the patients in the original cohort who successfully complete TB therapy can then be followed after treatment for 12 months to determine the proportion who experience disease relapse or death. As discussed above, we recommend following the rigorous prospective cohort methodology used by Velayutham et al. to determine post-treatment relapse rates [12]. Routine follow-up home visits of patients who have completed therapy every few months, to screen for symptoms and collect sputum samples for microscopy and mycobacterial culture on symptomatic patients, will minimized post-treatment loss to follow-up of patients, screen systematically for TB recurrence, and also carefully captured information on mortality [11].

Step E: Number of individuals with TB who reached health facilities and accessed a TB diagnostic test

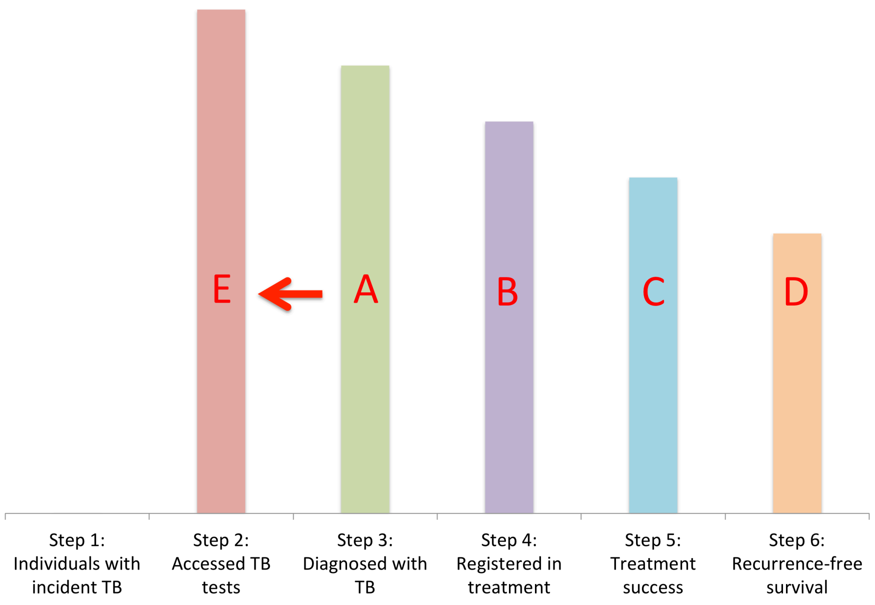

Figure 8: Step E in the cohort-based approach to constructing a TB care cascade

To estimate Step E for the cohort based approach (Figure 8), similar methodologies can be used as are described above for estimating Step E for the routine data approach. As described in detail above, different estimation approaches would be required for each form of TB.

Step F: Number of individuals with incident or prevalent TB in the population

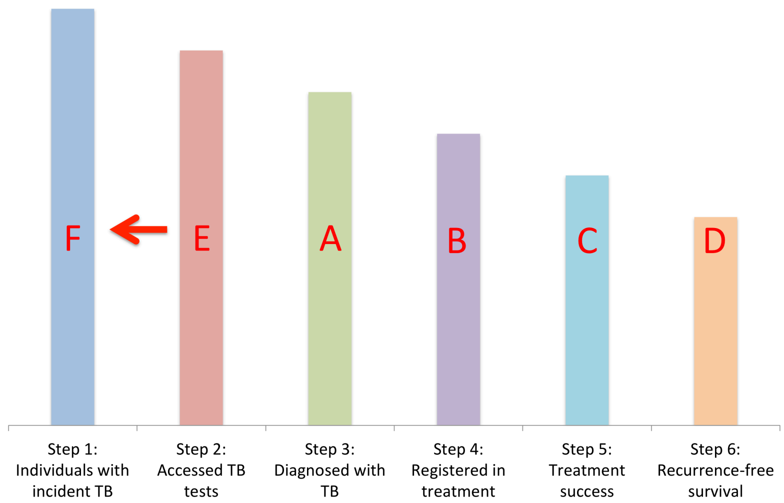

Figure 9: Step F in the cohort-based approach to constructing a TB care cascade

To estimate Step F for the cohort based approach (Figure 9), similar methodologies can be used as are described above for estimating Step F for the routine data approach.

References

1. Haber N, Pillay D, Porter K, Barnighausen T. Constructing the cascade of HIV care: methods for measurement. Curr Opin HIV AIDS. 2016;11(1):102-8. doi: 10.1097/coh.0000000000000212. PMID: 26545266.

2. Subbaraman R, Nathavitharana RR, Satyanarayana S, Pai M, Thomas BE, Chadha VK, et al. The Tuberculosis Cascade of Care in India's Public Sector: A Systematic Review and Meta-analysis. PLoS Med. 2016;13(10):e1002149. doi: 10.1371/journal.pmed.1002149. PMID: 27780217.

3. Naidoo P, Theron G, Rangaka MX, Chihota VN, Vaughan L, Brey ZO, et al. The South African Tuberculosis Care Cascade: Estimated Losses and Methodological Challenges. J Infect Dis. 2017;216(suppl_7):S702-S13. doi: 10.1093/infdis/jix335. PMID: 29117342.

4. Central TB Division. TB India 2017: Revised National TB Control Programme annual status report. New Delhi: Ministry of Health and Family Welfare, 2017.

5. Central TB Division. TB India 2016: Revised National TB Control Programme annual status report. New Delhi: Ministry of Health and Family Welfare, 2016.

6. Becerra MC, Appleton SC, Franke MF, Chalco K, Bayona J, Murray MB, et al. Recurrence after treatment for pulmonary multidrug-resistant tuberculosis. Clin Infect Dis. 2010;51(6):709-11. doi: 10.1086/655892. PMID: 20687835.
7. Cox H, Kebede Y, Allamuratova S, Ismailov G, Davletmuratova Z, Byrnes G, et al. Tuberculosis recurrence and mortality after successful treatment: Impact of drug resistance. PLoS Med. 2006;3(10):1836-43. doi: 10.1371/journal.pmed.0030384.
8. Gelmanova IY, Ahmad Khan F, Becerra MC, Zemlyanaya NA, Unakova IA, Andreev YG, et al. Low rates of recurrence after successful treatment of multidrug-resistant tuberculosis in Tomsk, Russia. Int J Tuberc Lung Dis. 2015;19(4):399-405. doi: 10.5588/ijtld.14.0415. PMID: 25859994.
9. Marx FM, Dunbar R, Enarson DA, Williams BG, Warren RM, van der Spuy GD, et al. The temporal dynamics of relapse and reinfection tuberculosis after successful treatment: a retrospective cohort study. Clin Infect Dis. 2014;58(12):1676-83. doi: 10.1093/cid/ciu186. PMID: 24647020.
10. Sadacharam K, Gopi PG, Chandrasekaran V, Eusuff SI, Subramani R, Santha T, et al. Status of smear-positive TB patients at 2-3 years after initiation of treatment under a DOTS programme. Indian J Tuberc. 2007;54(4):199-203. PMID: 18072535.
11. Thomas A, Gopi PG, Santha T, Chandrasekaran V, Subramani R, Selvakumar N, et al. Predictors of relapse among pulmonary tuberculosis patients treated in a DOTS programme in South India. Int J Tuberc Lung Dis. 2005;9(5):556-61. PMID: 15875929.
12. Velayutham B, Chadha VK, Singla N, Narang P, Gangadhar Rao V, Nair S, et al. Recurrence of tuberculosis among newly diagnosed sputum positive pulmonary tuberculosis patients treated under the Revised National Tuberculosis Control Programme, India: A multi-centric prospective study. PLoS One. 2018;13(7):e0200150. doi: 10.1371/journal.pone.0200150. PMID: 29979738.
13. Cox H, Dickson-Hall L, Ndjeka N, Van't Hoog A, Grant A, Cobelens F, et al. Delays and loss to follow-up before treatment of drug-resistant tuberculosis following implementation of Xpert MTB/RIF in South Africa: A retrospective cohort study. PLoS Med. 2017;14(2):e1002238. doi: 10.1371/journal.pmed.1002238. PMID: 28222095.
14. Kolappan C, Subramani R, Karunakaran K, Narayanan PR. Mortality of tuberculosis patients in Chennai, India. Bull World Health Organ. 2006;84(7):555-60. PMID: 16878229.
15. Kolappan C, Subramani R, Kumaraswami V, Santha T, Narayanan PR. Excess mortality and risk factors for mortality among a cohort of TB patients from rural south India. Int J Tuberc Lung Dis. 2008;12(1):81-6. PMID: 18173882.
16. MacPherson P, Houben R, Glynn JR, Corbett EL, Kranzer K. Pre-treatment loss to follow-up in tuberculosis patients in low- and lower-middle-income countries and high-burden countries: a systematic review and meta-analysis. Bull World Health Organ. 2014;92(2):126-38. doi: 10.2471/blt.13.124800. PMID: 24623906.
17. Uchenna OU, Chukwu JN, Onyeonoro UU, Oshi DC, Nwafor CC, Meka AO, et al. Pattern and magnitude of treatment delay among TB patients in five states
in southern Nigeria. Ann Trop Med Public Health. 2012;5(3):173-7.
18. Razia F, Ejaz Q, editors. Initial default common in tertiary care hospitals: descriptive study, Rawalpindi district, Pakistan. 41st Union World Conference on Lung Health; 11-15 November 2010; Berlin, Germany. Paris: International Union Against Tuberculosis and Lung Disease; 2011.
19. Buu TN, Lonnroth K, Quy HT. Initial defaulting in the National Tuberculosis Programme in Ho Chi Minh City, Vietnam: a survey of extent, reasons and alternative actions taken following default. Int J Tuberc Lung Dis. 2003;7(8):735-41. PMID: 12921149.
20. Korobitsyn A, Rajabov J, Norov O, Shekhov A, editors. Analysis of initial defaulters in selected districts in Tajikistan. 41st Union World Conference on Lung Health; 11-15 November 2010; Berlin, Germany. Paris: International Union Against Tuberculosis and Lung Disease; 2010.

21. Mase SR, Ramsay A, Ng V, Henry M, Hopewell PC, Cunningham J, et al. Yield of serial sputum specimen examinations in the diagnosis of pulmonary tuberculosis: a systematic review. Int J Tuberc Lung Dis. 2007;11(5):485-95. PMID: 17439669.
22. Steingart KR, Schiller I, Horne DJ, Pai M, Boehme CC, Dendukuri N. Xpert(R) MTB/RIF assay for pulmonary tuberculosis and rifampicin resistance in adults. Cochrane Database Syst Rev. 2014;1:Cd009593. doi: 10.1002/14651858.CD009593.pub3. PMID: 24448973.
23. Davis JL, Cattamanchi A, Cuevas LE, Hopewell PC, Steingart KR. Diagnostic accuracy of same-day microscopy versus standard microscopy for pulmonary tuberculosis: a systematic review and meta-analysis. Lancet Infect Dis. 2013;13(2):147-54. doi: 10.1016/s1473-3099(12)70232-3. PMID: 23099183.
24. Steingart KR, Henry M, Ng V, Hopewell PC, Ramsay A, Cunningham J, et al. Fluorescence versus conventional sputum smear microscopy for tuberculosis: a systematic review. Lancet Infect Dis. 2006;6(9):570-81. doi: 10.1016/s1473-3099(06)70578-3. PMID: 16931408.
25. Sachdeva KS, Raizada N, Sreenivas A, Van't Hoog AH, van den Hof S, Dewan PK, et al. Use of Xpert MTB/RIF in Decentralized Public Health Settings and Its Effect on Pulmonary TB and DR-TB Case Finding in India. PLoS One. 2015;10(5):e0126065. doi: 10.1371/journal.pone.0126065. PMID: 25996389.
26. Dorman SE, Schumacher SG, Alland D, Nabeta P, Armstrong DT, King B, et al. Xpert MTB/RIF Ultra for detection of Mycobacterium tuberculosis and rifampicin resistance: a prospective multicentre diagnostic accuracy study. Lancet Infect Dis. 2018;18(1):76-84. doi: 10.1016/s1473-3099(17)30691-6. PMID: 29198911.
27. Steingart KR, Ng V, Henry M, Hopewell PC, Ramsay A, Cunningham J, et al. Sputum processing methods to improve the sensitivity of smear microscopy for tuberculosis: a systematic review. Lancet Infect Dis. 2006;6(10):664-74. doi: 10.1016/s1473-3099(06)70602-8. PMID: 17008175.
28. Ministry of Health and Family Welfare. Report of the First National Anti-Tuberculosis Drug Resistance Survey (2014-16). New Delhi, India: Ministry of Health and Family Welfare, 2018.
29. Mwangwa F, Chamie G, Kwarisiima D, Ayieko J, Owaraganise A, Ruel TD, et al. Gaps in the Child Tuberculosis Care Cascade in 32 Rural Communities in Uganda and Kenya. J Clin Tuberc Other Mycobact Dis. 2017;9:24-9. doi: 10.1016/j.jctube.2017.10.003. PMID: 29291251.
30. Bates M, O'Grady J, Maeurer M, Tembo J, Chilukutu L, Chabala C, et al. Assessment of the Xpert MTB/RIF assay for diagnosis of tuberculosis with gastric lavage aspirates in children in sub-Saharan Africa: a prospective descriptive study. Lancet Infect Dis. 2013;13(1):36-42. doi: 10.1016/s1473-3099(12)70245-1. PMID: 23134697.
31. Detjen AK, DiNardo AR, Leyden J, Steingart KR, Menzies D, Schiller I, et al. Xpert MTB/RIF assay for the diagnosis of pulmonary tuberculosis in children: a systematic review and meta-analysis. Lancet Respir Med. 2015;3(6):451-61. doi: 10.1016/s2213-2600(15)00095-8. PMID: 25812968.
32. Onozaki I, Law I, Sismanidis C, Zignol M, Glaziou P, Floyd K. National tuberculosis prevalence surveys in Asia, 1990-2012: an overview of results and lessons learned. Trop Med Int Health. 2015;20(9):1128-45. doi: 10.1111/tmi.12534. PMID: 25943163.
33. Dhanaraj B, Papanna MK, Adinarayanan S, Vedachalam C, Sundaram V, Shanmugam S, et al. Prevalence and risk factors for adult pulmonary tuberculosis in a metropolitan city of South India. PLoS One. 2015;10(4):e0124260. doi: 10.1371/journal.pone.0124260. PMID: 25905900.
34. Haber N, Tanser F, Bor J, Naidu K, Mutevedzi T, Herbst K, et al. From HIV infection to therapeutic response: a population-based longitudinal HIV cascade-of-care study in KwaZulu-Natal, South Africa. Lancet HIV. 2017;4(5):E223-E30. doi: 10.1016/s2352-3018(16)30224-7. PMID: 28153470.
35. Arinaminpathy N, Batra D, Khaparde S, Vualnam T, Maheshwari N, Sharma L, et al. The number of privately treated tuberculosis cases in India: an estimation from drug sales data. Lancet Infect Dis. 2016;16:1255-60. doi: 10.1016/S1473-3099(16)30259-6. PMID: 27568356.
